# Peripheral Nerve Conduction and Anthropometric Characteristics in Elite American Football Players: An Exploratory Cross-Sectional Study

**DOI:** 10.3390/diagnostics16203343

**Published:** 2026-10-14

**Authors:** Mehtap Erdogan, Elif Sarıca Darol, Murat Alemdar

**Affiliations:** 1 Department of Anatomy, Faculty of Medicine, Sakarya University, 54290 Sakarya, Türkiye; 2 Department of Neurology, Sakarya Training and Research Hospital, 54100 Sakarya, Türkiye; dresdarol@hotmail.com; 3 Department of Neurology, Faculty of Medicine, Sakarya University, 54100 Sakarya, Türkiye; dr.alemdar@gmail.com

**Keywords:** American football, peripheral nerves, nerve conduction study, electrophysiology, anthropometry, sports injuries

## Abstract

**Background/Objectives**: American football exposes athletes to repeated collisions and intense physical loading. This exploratory study compared anthropometric characteristics and peripheral nerve conduction parameters between elite American football players and healthy sedentary men. **Methods**: Thirty-six elite male players and 39 sedentary male controls underwent anthropometric assessment and motor and sensory nerve conduction studies (NCSs) of the upper and lower extremities. Groups were compared with Welch’s *t*-test, and effect sizes (Hedges’ g) were reported with 95% confidence intervals. The false discovery rate (FDR) was controlled across all 110 NCS parameters, which were also analysed in linear models adjusted for age, for age, height and body mass index, and for age and local limb circumference. **Results**: Players had larger shoulder, upper-arm and forearm circumferences, whereas height and limb segment lengths were similar. After age adjustment and FDR control, players had longer accessory nerve motor latencies on both sides (right: 3.70 ± 0.86 vs. 2.74 ± 0.85 ms; left: 3.63 ± 0.94 vs. 2.69 ± 0.75 ms; both q < 0.001), a shorter left musculocutaneous motor latency and a faster left sural sensory conduction velocity. Median, ulnar, sural and tibial response amplitudes were lower in players, but these differences were attenuated after adjustment for age. **Conclusions**: Elite American football players differ from sedentary controls in a small number of nerve conduction parameters, most consistently in accessory nerve motor latency. Because the controls were sedentary and the design was cross-sectional, these differences cannot be attributed specifically to American football rather than to intensive athletic training in general. They should be regarded as hypothesis-generating findings that require independent replication.

## 1. Introduction

American football is one of the contact sports with a high risk of injury due to intense physical contact, sudden accelerations and decelerations, repeated collisions, and movements requiring high force. In this sport, athletes may be exposed to significant mechanical stresses on the musculoskeletal system and peripheral nerves during sprints, blocking, tackling, and direct contact with opponents. In particular, peripheral nerves in the shoulder girdle, upper extremities, and lower extremities can be affected by mechanisms such as stretching, compression, and repetitive microtraumas. Peripheral nerve injuries in athletes can develop as a result of acute trauma, as well as from repetitive mechanical stress and chronic microtraumas [1,2].

One of the significant neurological injuries seen in contact sports is the “stinger” or “burner” injury, which is particularly common in American football. These injuries are defined as transient neuropraxias associated with traction or compression mechanisms affecting the cervical nerve roots and/or the brachial plexus, and they typically present with neurological symptoms such as unilateral burning pain, paresthesia, sensory loss, and transient motor weakness [3,4]. In American football, stinger injuries represent a significant clinical problem, particularly at the professional and collegiate levels; NFL data indicate that these injuries occur with a certain frequency and typically do not result in lost time [4,5]. While most stingers present as short-term, self-limiting clinical conditions, the available evidence regarding the long-term neurological consequences of recurrent injuries and their effects on peripheral nerve function is limited [3,4].

Electrodiagnostic evaluation, particularly nerve conduction studies (NCSs), is an important method for assessing peripheral nerve function in athletes [2,6,7]. NCSs enable the objective assessment of the motor and sensory conduction properties of peripheral nerves and allows for the identification of changes in conduction velocities, latencies, and the amplitudes of motor and sensory action potentials [8]. In addition, NCSs can help detect electrophysiological abnormalities in athletes who do not have clinically apparent neurological symptoms [6,7].

The anthropometric and somatotype characteristics of American football players have been examined in previous studies [9]. It has been demonstrated that standard anthropometric measurements can be used to assess body composition in American football players [10]. However, the relationship between anthropometric characteristics and peripheral nerve conduction parameters in American football players has not been sufficiently elucidated. This relationship is also methodologically relevant, because limb dimensions, soft-tissue thickness and body mass can influence recorded response amplitudes and latencies independently of nerve function [11].

Previous studies examining peripheral nerve injuries in athletes have primarily focused on acute trauma, nerve compression, and clinically evident neurological findings [6,7,12]. However, it has not been sufficiently established whether long-term participation in American football is associated with peripheral nerve conduction parameters, particularly in the absence of clinically evident neurological symptoms. Repeated collisions, limb loading, and high-force athletic activities can expose peripheral nerves to compression, traction, and repetitive mechanical stress; it has been suggested that these mechanisms may be associated with electrophysiological changes in peripheral nerve function [2,13].

The aim of this exploratory study was to compare the anthropometric characteristics and peripheral nerve conduction parameters of elite American football players with those of healthy, sedentary controls. Motor and sensory nerve conduction parameters of the upper and lower extremities were examined with NCSs in order to describe electrophysiological differences between the groups and to generate hypotheses for future prospective studies, rather than to establish specific pathophysiological mechanisms.

## 2. Materials and Methods

### 2.1. Research Design and Participants

This study was designed as an exploratory cross-sectional study comparing peripheral nerve conduction parameters and anthropometric characteristics between American football players and a healthy control group. A total of 75 male volunteers were included in the study. The American football group consisted of 36 elite athletes who were actively competing in organized protective football leagues in Turkey and had at least three years of regular American football experience. All of the athletes were active players on the Sakarya Tatankaları team, which competes in the Turkish American Football 1st and 2nd Leagues. The control group consisted of 39 healthy male volunteers within a similar age range who did not engage in regular exercise and were not involved in elite-level athletic activities in any sport. Control participants were recruited from among university students.

Our study was initiated after it was approved by the Health Sciences Research Ethics Committee on 19 November 2024, under Decision No. E.421174. All participants were provided with detailed information about the purpose of the study and the procedures to be performed, and written informed consent was obtained from all participants prior to their participation in the study.

### 2.2. Inclusion and Exclusion Criteria

The American football group included male athletes aged 18–30 years who actively played American football and had at least three years of experience in the sport. The control group included healthy male volunteers aged 18–30 years who did not engage in regular exercise and were not involved in elite-level athletic activities in any sport.

Exclusion criteria for both groups were: diabetes mellitus or impaired glucose tolerance; thyroid disease; known vitamin B12 deficiency; chronic kidney or liver disease; alcohol or substance misuse; known polyneuropathy; cervical radiculopathy; previous peripheral nerve injury; clinically diagnosed carpal or cubital tunnel syndrome; previous fracture or surgery of the examined limbs; and use of medication with known neurotoxic effects.

No standardized neurological examination was performed; the absence of neurological symptoms was based on participants’ self-report.

### 2.3. Anthropometric Measurements

The participants’ age, height, and body weight were recorded. Height was measured to the nearest 0.1 cm with a stadiometer and body weight to the nearest 0.1 kg using a digital scale, with participants barefoot and in light clothing; body mass index (BMI) was calculated as weight/height^2^ (kg/m^2^). In addition, shoulder circumference, upper arm circumference, forearm circumference, thigh circumference, calf circumference, upper arm length, forearm length, total upper extremity length, thigh length, lower leg length, and total lower extremity length were measured with a non-extensible anthropometric tape. Limb measurements were recorded on one side and the same values were entered for both sides; right-side values are therefore reported. The following landmarks were used: upper arm length, acromiale to radiale; forearm length, radiale to stylion; total upper extremity length, acromiale to dactylion; thigh length, trochanterion to tibiale laterale; lower leg length, tibiale laterale to the floor; total lower extremity length, trochanterion to the floor; upper arm (relaxed) circumference, at the mid-acromiale–radiale level; forearm circumference, at the maximal girth; thigh circumference, at mid-thigh; calf circumference, at the maximal girth; and shoulder circumference, at the level of the greatest lateral protrusion of the deltoid muscles. All anthropometric measurements were performed by the same researcher in accordance with the criteria of the International Society for the Advancement of Kinanthropometry (ISAK) before the training at 10.00 a.m. [14,15].

On data verification, total upper extremity length was inconsistent with the sum of the upper arm and forearm segment lengths. The discrepancy was in opposite directions in the two groups: on average, total length was 5.8 cm longer than the sum in players but 4.2 cm shorter in controls. This indicates that the landmarks for this variable were not applied uniformly. Total upper extremity length was therefore excluded from the analysis, and upper-limb dimensions are reported using the segment lengths.

### 2.4. Nerve Conduction Studies (NCSs)

All electrophysiological evaluations were performed by the same experienced neurologist at the Electromyography (EMG) Laboratory of the Department of Neurology at Sakarya University Training and Research Hospital, using a Nicolet EDX system with Synergy software (Natus Medical, Galway, Ireland). Nerve conduction studies were carried out in accordance with the practice guidelines of the American Association of Electrodiagnostic Medicine (AAEM) and standard techniques [8,16]. Conventional procedures were used, with supramaximal percutaneous stimulation delivered by a handheld bipolar constant-current stimulator. Disposable disc electrodes were used for motor recordings and ring electrodes for digital sensory recordings. The studies were performed bilaterally in a quiet examination room kept at 23–25 °C, with participants supine and relaxed (seated for accessory nerve studies). In the lower extremity, the motor nerves (tibial and common peroneal nerves) and the sensory nerve (sural nerve) were evaluated; in the upper extremity, the median, ulnar, radial, axillary, musculocutaneous, and accessory nerves, as well as the medial and lateral antebrachial cutaneous nerves, were evaluated.

Supramaximal stimulation (current increased until the response amplitude no longer increased and then raised by a further 20–30%) was used for all motor and sensory studies, with a pulse duration of 0.1–0.2 ms. Filter settings were 2 Hz–10 kHz for motor and 20 Hz–2 kHz for sensory recordings. In line with the international standards of instrumentation for EMG [17,18], the display gain was set at 10 µV/division for sensory and 2 mV/division for motor recordings and was increased as required for F-wave recordings. The sweep speed was set at 1 ms/division for sensory, 2 ms/division for motor and 5–10 ms/division for F-wave recordings. Because temperature can materially affect latencies and conduction velocities [19], skin temperature was measured with a surface thermometer over the dorsum of the hand and foot before each study. It was maintained at ≥32 °C in the upper and ≥30 °C in the lower extremities, using warm water when necessary.

In motor nerve conduction studies, stimulation was applied at the wrist and elbow for the median nerve (recording from abductor pollicis brevis); at the wrist, below the elbow, and above the elbow for the ulnar nerve (abductor digiti minimi); at the forearm and elbow for the radial nerve (extensor indicis); at the ankle, fibular head, and popliteal fossa for the common peroneal nerve (extensor digitorum brevis); and at the ankle and popliteal fossa for the tibial nerve (abductor hallucis). Distal stimulation was applied at a fixed distance of 8 cm (upper limb) and 9 cm (lower limb) from the active recording electrode. The accessory nerve was stimulated at the posterior border of the sternocleidomastoid muscle (recording from the upper trapezius), and the axillary and musculocutaneous nerves at Erb’s point (recording from the deltoid and biceps brachii, respectively); for these nerves only single-site onset latencies and CMAP amplitudes were obtained. Onset latency, baseline-to-negative-peak CMAP amplitude and motor conduction velocity were recorded; motor conduction velocities were calculated by dividing the distance between the distal and proximal stimulation sites, measured with a tape along the nerve course, by the difference between the corresponding latencies. In the tibial nerve studies, the F–M latency was also obtained from 10–20 supramaximal stimuli. Sensory studies were performed antidromically: for the median nerve between the wrist and the second digit, for the ulnar nerve between the wrist and the fifth digit (14 cm), for the superficial radial nerve between the forearm and the extensor pollicis longus (EPL) tendon (10 cm), for the sural nerve between the mid-posterior calf and the lateral malleolus (14 cm), and for the medial and lateral antebrachial cutaneous nerves between the elbow and the mid-forearm (12 cm). Onset latency (median, ulnar and radial nerves) or peak latency (sural and antebrachial cutaneous nerves), baseline-to-peak SNAP amplitude, and sensory conduction velocity were recorded.

### 2.5. Data Quality Control

All NCS values were checked in two steps, using the pooled data of both groups. First, values that were extreme relative to the pooled distribution (robust z-score > 6, based on the median and median absolute deviation), internally inconsistent or missing were checked against the original EMG reports. In this step, 33 transcription errors were corrected and 13 empty fields were completed; all corrected and completed values were taken directly from the original EMG reports. Second, four rules were applied algorithmically to all 8250 NCS cells of the verified dataset (8196 recorded values): (1) values ≤ 0 (0 had been used as a missing-value code); (2) conduction velocities < 20 m/s or >100 m/s; (3) latencies or amplitudes more than five times the pooled median of the respective parameter; and (4) an internal-consistency check. For rule 4, the conduction distance implied by each recording (velocity × onset or peak latency for sensory recordings; velocity × latency difference for motor segments) was compared with the pooled median of that quantity, and values below half or above twice the median were considered inconsistent.

Values meeting rules 1–3 were regarded as measurement or transcription errors and set to missing (10 values, 0.1% of all NCS values, from 8 participants). Every recording identified by rule 4 also contained one of these values, so rule 4 led to no additional exclusions. Values that were markedly abnormal but did not meet the rules—such as very low SNAP amplitudes with normal latencies—were retained as potentially genuine abnormal observations. The rules depended only on the values themselves and were applied identically to both groups, without reference to the results of the between-group comparisons. The rules were formalized during the revision of this manuscript and were applied retrospectively to the complete dataset. The checks against the EMG reports were not blinded to group, because the participant codes identified the group. All values that could not be verified against the EMG reports, and the decision taken for each, are listed in Supplementary Table S2. Two sensitivity analyses were performed: one retaining all values exactly as recorded, and a stricter one that also excluded the 12 abnormal but possible values that could not be verified (Supplementary Table S4).

### 2.6. Statistical Analysis

This study was exploratory, and no single primary electrophysiological outcome was specified a priori. All NCS parameters were therefore treated as one family of outcomes.

Analyses were performed with Python 3.11 using SciPy 1.17 and statsmodels 0.15. Descriptive statistics are presented as mean ± standard deviation (SD) and, in Supplementary Table S1, as median (interquartile range). Normality was assessed with the Shapiro–Wilk test and Q–Q plots, and homogeneity of variances with the Brown–Forsythe (median-centred Levene) test. Because variances were frequently unequal and some variables deviated from normality, between-group comparisons were performed with Welch’s *t*-test, which does not assume equal variances [20]; the Mann–Whitney U test was used as a sensitivity analysis. For each comparison, the mean difference (players minus controls) with its 95% confidence interval (CI) and Hedges’ g with its 95% CI were reported as measures of magnitude and precision; g values of approximately 0.2, 0.5 and 0.8 were interpreted as small, medium and large, respectively [21].

To address multiplicity, the Benjamini–Hochberg procedure was applied across all 110 NCS comparisons and q-values (FDR-adjusted *p*-values) are reported [22,23]; the anthropometric variables were treated as a separate family. Findings with q < 0.05 were regarded as robust to multiple testing, and findings with *p* < 0.05 but q ≥ 0.05 as nominal and hypothesis-generating.

Because the groups differed in age and body size, every NCS parameter was also analysed with linear regression (analysis of covariance), with group (football player = 1, control = 0) as the independent variable. Three covariate sets were defined: Model 1, age; Model 2, age, height and BMI; and Model 3, age and the circumference of the limb segment over which the response was recorded (calf circumference for lower-extremity nerves, shoulder circumference for the accessory nerve, upper-arm circumference for the axillary and musculocutaneous nerves, and forearm circumference for the remaining upper-extremity nerves). The regression coefficient β thus represents the adjusted mean difference between players and controls. FDR control was applied separately within each model. Complete results are given in Supplementary Table S1 (Model 1) and Supplementary Table S3 (Models 2 and 3). Table 1 shows all three models for parameters with *p* < 0.05 in the unadjusted or Model 1 analysis. All tests were two-sided with α = 0.05.

## 3. Results

### 3.1. Participants and Anthropometric Characteristics

The study included 36 American football players and 39 healthy controls. Players were older than controls (22.2 ± 2.8 vs. 20.1 ± 1.5 years; g = 0.98, *p* < 0.001); therefore, all NCS parameters were additionally analysed with age-adjusted models (Section 3.5). After data verification, 10 NCS values (0.1%) met the predefined exclusion rules and were set to missing (Section 2.5; Supplementary Table S2); the number of participants contributing to each comparison is shown in Table 2, Table 3 and Table 4 when it was lower than the full group size.

Body weight was nominally higher in players than in controls (88.2 ± 17.8 vs. 80.5 ± 14.2 kg; g = 0.48, *p* = 0.043, q = 0.111), whereas height (1.81 ± 0.07 vs. 1.80 ± 0.07 m; *p* = 0.455) and BMI (26.8 ± 4.8 vs. 24.8 ± 4.0 kg/m^2^; g = 0.44, *p* = 0.059) did not differ significantly (Table 5). Players had markedly greater shoulder circumference (53.7 ± 5.5 vs. 42.8 ± 3.1 cm; g = 2.44, *p* < 0.001, q < 0.001) and larger upper-arm (33.8 ± 2.8 vs. 30.8 ± 4.5 cm; g = 0.78, *p* < 0.001, q = 0.004) and forearm circumferences (28.9 ± 2.2 vs. 26.3 ± 3.1 cm; g = 0.93, *p* < 0.001, q < 0.001). In contrast, upper arm and forearm lengths, total lower extremity length, thigh length, and thigh and calf circumferences did not differ significantly between the groups (all *p* > 0.05; Table 5). Lower leg length was slightly greater in players (52.7 ± 4.4 vs. 50.5 ± 3.7 cm; g = 0.53, *p* = 0.023, q = 0.076). Overall, the groups differed mainly in upper-body circumferences and, to a lesser extent, in body mass, rather than in height or limb segment lengths.

### 3.2. Lower-Extremity Nerve Conduction Studies

In the lower extremities (Table 2), right peroneal motor conduction velocity in the distal segment was higher in players (50.8 ± 5.3 vs. 47.7 ± 4.2 m/s; g = 0.64, *p* = 0.007, q = 0.070) and right peroneal motor latency at the fibular head was shorter (10.76 ± 0.99 vs. 11.34 ± 1.07 ms; g = −0.56, *p* = 0.017, q = 0.092). Left tibial motor latency at the popliteal fossa was shorter in players (12.44 ± 1.22 vs. 13.16 ± 1.42 ms; g = −0.54, *p* = 0.020, q = 0.099). Left sural sensory conduction velocity was also higher in players (55.9 ± 7.6 vs. 51.3 ± 7.7 m/s; g = 0.59, *p* = 0.012, q = 0.087). None of these unadjusted differences remained significant after FDR control. Left tibial motor conduction velocity (49.4 ± 4.5 vs. 47.6 ± 4.3 m/s; *p* = 0.084) and left peroneal conduction velocities and latencies did not differ significantly between the groups.

Tibial CMAP amplitudes were lower in players on both sides. At the ankle, amplitudes were lower on the right (8.44 ± 2.38 vs. 10.49 ± 3.37 mV; g = −0.69, *p* = 0.003, q = 0.043) and on the left (8.73 ± 2.52 vs. 10.61 ± 3.86 mV; g = −0.57, *p* = 0.014, q = 0.092). At the popliteal fossa, they were lower on the right (7.00 ± 1.97 vs. 8.97 ± 3.24 mV; g = −0.72, *p* = 0.002, q = 0.035) and on the left (7.00 ± 1.98 vs. 9.03 ± 3.77 mV; g = −0.66, *p* = 0.005, q = 0.056). Peroneal CMAP amplitudes did not differ between the groups (Table 2).

Sural SNAP amplitudes were lower in players bilaterally (right: 17.58 ± 6.10 vs. 21.88 ± 9.37 µV; g = −0.53, *p* = 0.021, q = 0.099; left: 17.33 ± 5.83 vs. 21.77 ± 8.85 µV; g = −0.58, *p* = 0.012, q = 0.087), while sural peak latencies were similar and sensory conduction velocities were similar (right) or higher (left) in players (Table 2).

### 3.3. Shoulder-Girdle and Proximal Upper-Extremity Nerve Conduction Studies

Accessory nerve motor latency was longer in players on both sides, with large effect sizes (right: 3.70 ± 0.86 vs. 2.74 ± 0.85 ms; g = 1.11, *p* < 0.001, q < 0.001; left: 3.63 ± 0.94 vs. 2.69 ± 0.75 ms; g = 1.09, *p* < 0.001, q < 0.001; Table 3). One player’s right-sided value of 31.7 ms, more than ten times the pooled median, was excluded according to the predefined rules (Section 2.5). Right axillary motor latency was longer in players (3.34 ± 1.07 vs. 2.81 ± 0.50 ms; g = 0.63, *p* = 0.009, q = 0.080), whereas left axillary latency did not differ (3.16 ± 0.70 vs. 3.00 ± 0.51 ms; *p* = 0.268). Accessory, axillary and musculocutaneous CMAP amplitudes did not differ between the groups.

Musculocutaneous motor latencies to the biceps brachii were shorter in players on both sides (right: 2.80 ± 0.76 vs. 3.29 ± 0.93 ms; g = −0.56, *p* = 0.015, q = 0.092; left: 2.74 ± 0.83 vs. 3.38 ± 0.88 ms; g = −0.75, *p* = 0.002, q = 0.031; Table 3). Medial and lateral antebrachial cutaneous SNAP amplitudes, conduction velocities and peak latencies did not differ between the groups (all *p* > 0.05; Table 3).

### 3.4. Distal Upper-Extremity Nerve Conduction Studies

Median and ulnar SNAP amplitudes were lower in players on both sides (Table 4). Median SNAP amplitudes were lower on the right (41.80 ± 13.71 vs. 52.77 ± 12.09 µV; g = −0.84, *p* < 0.001, q = 0.011) and on the left (43.29 ± 15.69 vs. 50.62 ± 12.97 µV; g = −0.51, *p* = 0.032, q = 0.139). Ulnar SNAP amplitudes were lower on the right (32.53 ± 11.67 vs. 44.30 ± 13.09 µV; g = −0.94, *p* < 0.001, q = 0.004) and on the left (32.46 ± 12.48 vs. 42.77 ± 11.67 µV; g = −0.85, *p* < 0.001, q = 0.011). The corresponding sensory conduction velocities and onset latencies did not differ significantly (all *p* > 0.05). Superficial radial sensory parameters did not differ between the groups.

Ulnar motor latencies below and above the elbow were shorter in players on both sides (Table 4). On the right, latencies were shorter below the elbow (6.07 ± 0.64 vs. 6.56 ± 0.84 ms; g = −0.66, *p* = 0.007, q = 0.070) and above the elbow (7.69 ± 0.76 vs. 8.24 ± 0.91 ms; g = −0.64, *p* = 0.008, q = 0.074). On the left, they were shorter below the elbow (6.17 ± 0.59 vs. 6.60 ± 1.04 ms; g = −0.50, *p* = 0.030, q = 0.139) and above the elbow (7.87 ± 0.73 vs. 8.36 ± 1.01 ms; g = −0.55, *p* = 0.018, q = 0.096). Left median CMAP amplitudes were lower in players at the wrist (8.92 ± 2.16 vs. 10.58 ± 3.59 mV; g = −0.55, *p* = 0.017, q = 0.092) and elbow (8.43 ± 2.00 vs. 10.00 ± 3.31 mV; g = −0.56, *p* = 0.015, q = 0.092), whereas right median motor parameters did not differ. Right radial motor latency at the elbow did not differ between the groups with Welch’s *t*-test (5.63 ± 0.61 vs. 5.54 ± 1.56 ms; *p* = 0.742), but was longer in players with the Mann–Whitney test (*p* = 0.007). This discrepancy reflects two controls with markedly prolonged right radial latencies (11.2 and 12.3 ms at the elbow), which were retained as potentially genuine values. Left radial latency at the elbow and right radial motor conduction velocity did not differ between the groups.

### 3.5. Adjusted Analyses and Sensitivity Analyses

In age-adjusted models (Model 1) with FDR control, four parameters differed robustly between the groups (Table 1). Players had longer accessory nerve motor latencies on the right (β = 1.17 ms, 95% CI 0.74 to 1.60, *p* < 0.001, q < 0.001) and left (β = 1.29 ms, 95% CI 0.89 to 1.69, *p* < 0.001, q < 0.001), a shorter left musculocutaneous motor latency (β = −0.82 ms, 95% CI −1.25 to −0.38, *p* < 0.001, q = 0.012) and a higher left sural sensory conduction velocity (β = 6.9 m/s, 95% CI 3.2 to 10.7, *p* < 0.001, q = 0.012). Several differences remained nominally significant after age adjustment but did not survive FDR control, including the lower right ulnar SNAP amplitude (β = −9.40 µV, 95% CI −15.74 to −3.07, *p* = 0.004, q = 0.087) and the higher right peroneal distal motor conduction velocity (β = 3.6 m/s, 95% CI 1.1 to 6.1, *p* = 0.005, q = 0.087).

Several unadjusted differences were attenuated after adjustment for age: the right sural SNAP amplitude (β = −3.82 µV, 95% CI −7.95 to 0.31, *p* = 0.069), left median SNAP amplitude (β = −2.43 µV, 95% CI −9.42 to 4.56, *p* = 0.490), left median CMAP amplitudes at the wrist (β = −1.21 mV, 95% CI −2.75 to 0.32, *p* = 0.119) and elbow (β = −1.12 mV, 95% CI −2.53 to 0.30, *p* = 0.120) and left tibial CMAP amplitude at the ankle (β = −1.43 mV, 95% CI −3.12 to 0.26, *p* = 0.096) no longer reached nominal significance. Conversely, left tibial motor conduction velocity became nominally higher in players after age adjustment (β = 2.3 m/s, 95% CI 0.1 to 4.6, *p* = 0.044). The remaining amplitude differences in the median, ulnar, sural and tibial nerves did not survive FDR control (Table 1). Additional adjustment for height and BMI (Model 2) or for local limb circumference (Model 3) gave estimates of similar direction and magnitude (Table 1; Supplementary Table S3). In Model 3, the left accessory motor latency and left sural sensory conduction velocity remained significant after FDR control, whereas the right accessory latency (*p* = 0.002) and left musculocutaneous latency (*p* = 0.004) remained nominally significant. In the Mann–Whitney analysis, the direction of all differences was unchanged and all robust findings remained significant (Supplementary Table S1). The nominal differences in right sural SNAP amplitude, left median SNAP and CMAP amplitudes and left tibial ankle CMAP amplitude were not significant with this test, whereas left radial sensory onset latency (*p* = 0.043) and right radial motor latency at the elbow (*p* = 0.007) were significant only with this test.

The sensitivity analyses of data handling gave consistent results for the principal findings (Supplementary Table S4). When all values were analysed exactly as recorded, the left accessory latency, left musculocutaneous latency and left sural sensory conduction velocity remained robust in Model 1. The right accessory latency difference was no longer robust (q = 0.761), because this analysis included the single recorded value of 31.7 ms. When the 12 abnormal but possible values were also excluded, the right ulnar SNAP amplitude (q = 0.040) and right radial motor latency at the elbow (q = 0.020) also became robust. The radial difference therefore depended entirely on two extreme control values and is not interpreted further.

In summary, compared with sedentary controls, the football players showed (1) longer accessory nerve motor latencies on both sides, the most consistent difference across analyses; (2) a shorter musculocutaneous latency, robust on the left and nominal on the right; (3) a faster left sural sensory conduction velocity, without a corresponding difference on the right; (4) lower median, ulnar and sural SNAP amplitudes and lower tibial CMAP amplitudes, which were attenuated after adjustment for age; and (5) similar or slightly shorter motor latencies and similar or slightly faster conduction velocities in several distal motor nerves. These are group-level electrophysiological differences; individual values were not classified as normal or abnormal against reference limits.

## 4. Discussion

In this exploratory cross-sectional study, anthropometric characteristics and peripheral nerve conduction parameters of elite American football players were compared with those of healthy, sedentary men. Players had larger upper-body circumferences and nominally higher body mass, and several electrophysiological differences were observed. The differences that were robust to age adjustment and control of the false discovery rate were longer accessory nerve motor latencies on both sides, a shorter left musculocutaneous latency and a faster left sural sensory conduction velocity. Except for the right accessory latency, which depended on the exclusion of one recorded value of 31.7 ms, these findings were also robust in sensitivity analyses of data handling. Other differences, including lower median, ulnar, sural and tibial response amplitudes and faster peroneal motor conduction, were nominal and should be interpreted with caution. NCSs measure conduction properties rather than nerve structure, and this was a single cross-sectional assessment. The discussion below therefore distinguishes between the observed differences, the electrophysiological patterns they may be compatible with, and possible explanations that remain to be tested. Chronic biomechanical loading in contact sports has been proposed to affect both the musculoskeletal and the peripheral nervous systems [2,3]. Because the controls were sedentary, however, none of the present findings can be attributed specifically to American football rather than to intensive athletic training in general.

Players had greater shoulder, upper-arm and forearm circumferences than controls. Circumferences reflect the combined contribution of muscle, adipose tissue, bone and other soft tissues; therefore, we describe these findings as greater upper-extremity anthropometric dimensions rather than muscular hypertrophy, which would require direct measurement of muscle mass (e.g., by DXA, ultrasound or MRI). Such dimensions are nevertheless consistent with the resistance training and upper-body force production required for blocking and tackling, and previous studies have described physiological and neuromuscular adaptations over a competitive American football season [24,25]. Height and limb segment lengths did not differ between the groups. Total upper extremity length was excluded because the landmarks had not been applied consistently in the two groups. Larger muscle volume and connective tissue thickness may alter the mechanical environment of peripheral nerves [26], but they can also influence NCS recordings directly, as discussed below.

Lower SNAP amplitudes with similar sensory conduction velocities and latencies were observed in the median, ulnar and sural nerves. Electrophysiologically, a reduction in amplitude without slowing is the pattern that would be expected with a reduced number of conducting sensory axons rather than demyelination [8,27]; our findings are therefore compatible with, but do not demonstrate, mild axonal involvement. Several non-neural explanations must be considered. SNAP and CMAP amplitudes are influenced by soft-tissue thickness, electrode-to-nerve distance, digit circumference, skin thickness and callus formation, and body mass [11]; these factors are likely to differ between athletes who repeatedly grip, block and tackle and sedentary individuals. The amplitude differences were attenuated after adjustment for age and, to a lesser extent, for BMI and local limb circumference, and none remained robust to FDR control in age-adjusted models. One player had markedly low median and ulnar SNAP amplitudes on both sides, with onset latencies in the normal range; these values were retained as potentially genuine, but their clinical meaning cannot be determined without a neurological examination. Repeated collisions and high-energy contact could expose long sensory fibers to mechanical stress [2,28], but the present cross-sectional data cannot distinguish such effects from anatomical, technical or selection-related factors, and they should not be taken as evidence of axonal loss.

The most consistent finding was a longer accessory nerve motor latency on both sides. Its biological meaning is uncertain, and several explanations are possible. Mechanical loading of the neck and shoulder girdle during tackling—including forced lateral flexion of the neck and depression of the shoulder, the mechanism implicated in stinger injuries [3,4,5]—could affect proximal conduction. However, the cervical roots and brachial plexus were not evaluated directly, no needle EMG or imaging was performed, and previous stinger episodes were not analysed. The findings therefore cannot be interpreted as evidence of brachial plexus traction injury. Technical and anatomical explanations are equally plausible. The accessory nerve was stimulated at a fixed anatomical site, and latencies were not normalized to nerve length; greater neck and shoulder dimensions may therefore lengthen the conduction path and increase the depth of the nerve below the stimulator. Consistent with this, adjustment for shoulder circumference attenuated the right-sided difference, although the left-sided difference persisted. Conversely, musculocutaneous latencies were shorter in players, and ulnar motor latencies below and above the elbow were also shorter. These findings argue against a uniform pattern of proximal slowing. They may relate to recording geometry over a larger biceps muscle, differences in stimulation depth or distance, or physiological differences between trained and sedentary individuals. We therefore do not favour any single mechanism, and the accessory nerve finding requires independent replication.

Motor conduction velocities were similar or slightly faster in players in several nerves, and the left sural sensory conduction velocity was higher in players after age adjustment. The sural finding was not observed on the right side, and its biological meaning is unclear. Regular high-intensity exercise has been associated with changes in myelin, Schwann cell activity and nerve regeneration in experimental studies [29,30], so training-related adaptation is one possible explanation. Differences in limb temperature, distance measurement and age can also produce velocity differences of a few metres per second [11,19]. The present data cannot distinguish between these explanations. As with the accessory nerve finding, we do not favour any single mechanism, and this observation should be replicated independently before it is interpreted biologically.

It should be emphasized that the control group was sedentary. The design therefore cannot determine whether any of the observed differences are specific to American football, with its repeated collisions and traction forces, or are shared with other forms of intensive athletic training, resistance exercise, or sport-related selection. Differences between athletes and sedentary individuals in body composition, physical fitness and limb temperature could contribute to all of the findings reported here. A control group of non-contact athletes with comparable age and training exposure would be required to address this question.

None of the participants reported neurological symptoms. However, this was based on self-report, and no formal neurological examination was performed. The absence of clinically detectable neurological abnormalities therefore cannot be assumed, and unrecognized neurological conditions in individual participants cannot be excluded. Group-level differences do not indicate clinical abnormality in individual athletes, and their functional significance is unknown. Nerve conduction studies may complement the clinical evaluation of contact-sport athletes with symptoms, but longitudinal evidence is needed before routine or periodic NCSs can be recommended for asymptomatic athletes. Future studies should follow athletes prospectively across the preseason, competitive season and postseason and should include non-contact athletic controls. They should also record playing position, career duration, cumulative exposure, previous stinger episodes and limb dominance, and combine NCSs with a standardized neurological examination, needle EMG and nerve ultrasound or MRI.

## 5. Conclusions

Compared with healthy sedentary men, elite American football players had larger upper-body circumferences and differed in a small number of nerve conduction parameters. The most consistent difference was a longer accessory nerve motor latency on both sides. A shorter left musculocutaneous latency and a faster left sural sensory conduction velocity were also robust to adjustment and to correction for multiple comparisons. Lower median, ulnar, sural and tibial amplitudes were observed, but most of these differences were attenuated after adjustment for age. Because the controls were sedentary, the differences cannot be attributed specifically to American football rather than to intensive athletic training in general. Together with the cross-sectional design and the exploratory analyses, this means that the findings should be regarded as hypothesis-generating, not as evidence of nerve injury or neural adaptation. Independent replication in prospective, position-specific studies with non-contact athletic controls is needed.

## 6. Limitations

Several limitations should be considered when interpreting these findings. First, the cross-sectional design does not allow causal inference, and the study cannot determine whether the observed differences are stable, progressive or reversible. Second, the study was exploratory, with a large number of comparisons and no prespecified primary outcome; although FDR control was applied, nominal findings may be false positives and all findings require replication. Third, the sample was relatively small and recruited from a single team and centre, which limits precision and generalizability. Fourth, players were older than controls; although all NCS parameters were analysed with age-adjusted models, residual confounding by age cannot be excluded. Fifth, the groups differed in body mass and limb circumferences, which can influence response amplitudes and latencies through soft-tissue and recording-geometry effects; adjustment for BMI and limb circumference may not fully capture these factors, and residual anthropometric confounding is possible. Sixth, and importantly, the control group was sedentary. The design therefore cannot separate effects specific to American football from those of intensive athletic training in general, resistance exercise, or sport-related selection. Seventh, the electrophysiological findings were not supplemented by needle EMG, nerve ultrasound, MRI or follow-up assessments, so their structural and functional correlates remain unknown. Eighth, playing position, career duration, training load, collision exposure, limb dominance and previous stinger/burner episodes were not analysed. Ninth, ten physiologically implausible values that could not be verified against the original EMG reports were set to missing. Apart from the right accessory latency, which depended on the exclusion of one value of 31.7 ms, the principal findings did not depend on this decision; one difference that was robust only in the stricter analysis (right radial motor latency at the elbow) depended entirely on the handling of two extreme control values. In addition, limb anthropometric measurements were recorded on one side only, and total upper extremity length had to be excluded because of inconsistent measurement. Tenth, no standardized neurological examination was performed, and the absence of neurological symptoms was based on self-report; unrecognized neurological conditions in individual participants therefore cannot be excluded. Finally, the study was conducted in a Turkish population, and the findings may not be generalizable to other populations or competitive levels.

## Figures and Tables

**Table 1:** Adjusted differences between American football players and controls for nerve conduction parameters with *p* < 0.05 in unadjusted or age-adjusted analyses.

NCS Parameter	Model 1: β (95% CI)	Model 1: *p* (q)	Model 2: β (95% CI)	Model 2: *p* (q)	Model 3: β (95% CI)	Model 3: *p* (q)
Right common peroneal nerve: MCV, distal segment (m/s)	3.6 (1.1 to 6.1)	0.005 (0.087)	4.0 (1.5 to 6.4)	0.002 (0.044)	3.6 (1.1 to 6.1)	0.006 (0.127)
Right common peroneal nerve: Motor latency, fibular head (ms)	−0.69 (−1.22 to −0.16)	0.012 (0.116)	−0.79 (−1.30 to −0.27)	0.004 (0.065)	−0.71 (−1.25 to −0.18)	0.009 (0.148)
Right tibial nerve: CMAP amplitude, ankle (mV)	−1.68 (−3.19 to −0.17)	0.030 (0.182)	−1.60 (−3.14 to −0.06)	0.042 (0.209)	−1.58 (−3.07 to −0.08)	0.039 (0.227)
Right tibial nerve: CMAP amplitude, popliteal fossa (mV)	−1.73 (−3.14 to −0.32)	0.017 (0.141)	−1.61 (−3.04 to −0.18)	0.028 (0.180)	−1.65 (−3.05 to −0.24)	0.023 (0.203)
Right sural nerve: SNAP amplitude (µV)	−3.82 (−7.95 to 0.31)	0.069 (0.305)	−3.70 (−7.80 to 0.41)	0.077 (0.283)	−3.99 (−8.13 to 0.16)	0.059 (0.296)
Left common peroneal nerve: MCV, distal segment (m/s)	2.7 (0.2 to 5.3)	0.037 (0.194)	3.0 (0.5 to 5.6)	0.020 (0.149)	2.7 (0.1 to 5.3)	0.039 (0.227)
Left tibial nerve: CMAP amplitude, ankle (mV)	−1.43 (−3.12 to 0.26)	0.096 (0.340)	−1.28 (−2.98 to 0.41)	0.136 (0.410)	−1.35 (−3.04 to 0.35)	0.117 (0.396)
Left tibial nerve: CMAP amplitude, popliteal fossa (mV)	−1.68 (−3.25 to −0.11)	0.037 (0.194)	−1.52 (−3.09 to 0.06)	0.058 (0.255)	−1.60 (−3.17 to −0.03)	0.046 (0.255)
Left tibial nerve: MCV (m/s)	2.3 (0.1 to 4.6)	0.044 (0.215)	2.7 (0.5 to 5.0)	0.019 (0.149)	2.4 (0.2 to 4.7)	0.037 (0.227)
Left tibial nerve: Motor latency, popliteal fossa (ms)	−0.81 (−1.50 to −0.12)	0.021 (0.162)	−0.89 (−1.58 to −0.20)	0.012 (0.104)	−0.84 (−1.53 to −0.15)	0.018 (0.199)
Left sural nerve: SNAP amplitude (µV)	−4.55 (−8.47 to −0.63)	0.024 (0.162)	−4.20 (−8.09 to −0.30)	0.035 (0.195)	−4.53 (−8.49 to −0.56)	0.026 (0.203)
Left sural nerve: SCV (m/s)	6.9 (3.2 to 10.7)	<0.001 (0.012)	7.0 (3.3 to 10.8)	<0.001 (0.014)	6.9 (3.1 to 10.7)	<0.001 (0.028)
Right accessory nerve: Motor latency (ms)	1.17 (0.74 to 1.60)	<0.001 (<0.001)	1.12 (0.69 to 1.55)	<0.001 (<0.001)	1.01 (0.38 to 1.63)	0.002 (0.077)
Right axillary nerve: Motor latency (ms)	0.59 (0.16 to 1.02)	0.008 (0.087)	0.56 (0.13 to 1.00)	0.011 (0.104)	0.49 (0.03 to 0.94)	0.036 (0.227)
Right musculocutaneous nerve: Motor latency (ms)	−0.62 (−1.05 to −0.18)	0.006 (0.087)	−0.59 (−1.03 to −0.15)	0.009 (0.104)	−0.53 (−1.00 to −0.07)	0.026 (0.203)
Left accessory nerve: Motor latency (ms)	1.29 (0.89 to 1.69)	<0.001 (<0.001)	1.23 (0.84 to 1.62)	<0.001 (<0.001)	1.21 (0.63 to 1.80)	<0.001 (0.011)
Left musculocutaneous nerve: Motor latency (ms)	−0.82 (−1.25 to −0.38)	<0.001 (0.012)	−0.79 (−1.23 to −0.36)	<0.001 (0.015)	−0.69 (−1.15 to −0.23)	0.004 (0.097)
Right median nerve: SNAP amplitude (2nd digit) (µV)	−8.17 (−14.70 to −1.64)	0.015 (0.137)	−6.75 (−12.90 to −0.59)	0.032 (0.195)	−7.66 (−14.76 to −0.56)	0.035 (0.227)
Right ulnar nerve: SNAP amplitude (5th digit) (µV)	−9.40 (−15.74 to −3.07)	0.004 (0.087)	−8.30 (−14.50 to −2.09)	0.009 (0.104)	−8.53 (−15.40 to −1.65)	0.016 (0.193)
Right ulnar nerve: MCV (m/s)	4.0 (0.5 to 7.4)	0.024 (0.162)	4.4 (1.0 to 7.9)	0.012 (0.104)	4.8 (1.1 to 8.5)	0.012 (0.165)
Right ulnar nerve: Motor latency, below elbow (ms)	−0.54 (−0.94 to −0.15)	0.008 (0.087)	−0.54 (−0.95 to −0.14)	0.010 (0.104)	−0.51 (−0.94 to −0.08)	0.022 (0.203)
Right ulnar nerve: Motor latency, above elbow (ms)	−0.62 (−1.07 to −0.18)	0.006 (0.087)	−0.66 (−1.11 to −0.21)	0.005 (0.074)	−0.66 (−1.14 to −0.17)	0.009 (0.148)
Left median nerve: SNAP amplitude (2nd digit) (µV)	−2.43 (−9.42 to 4.56)	0.490 (0.781)	−1.49 (−8.42 to 5.45)	0.670 (0.896)	−2.42 (−10.01 to 5.17)	0.526 (0.824)
Left median nerve: CMAP amplitude, wrist (mV)	−1.21 (−2.75 to 0.32)	0.119 (0.365)	−0.97 (−2.47 to 0.54)	0.204 (0.495)	−0.83 (−2.48 to 0.81)	0.317 (0.645)
Left median nerve: CMAP amplitude, elbow (mV)	−1.12 (−2.53 to 0.30)	0.120 (0.365)	−0.94 (−2.33 to 0.46)	0.185 (0.463)	−0.83 (−2.36 to 0.69)	0.280 (0.601)
Left ulnar nerve: SNAP amplitude (5th digit) (µV)	−6.73 (−12.71 to −0.75)	0.028 (0.181)	−5.64 (−11.47 to 0.19)	0.058 (0.255)	−5.28 (−11.71 to 1.15)	0.106 (0.396)
Left ulnar nerve: Motor latency, below elbow (ms)	−0.45 (−0.90 to −0.01)	0.045 (0.215)	−0.47 (−0.92 to −0.02)	0.042 (0.209)	−0.44 (−0.92 to 0.04)	0.072 (0.331)
Left ulnar nerve: Motor latency, above elbow (ms)	−0.49 (−0.96 to −0.03)	0.036 (0.194)	−0.53 (−1.00 to −0.07)	0.026 (0.177)	−0.50 (−1.00 to 0.00)	0.051 (0.269)

β = adjusted mean difference, football players minus controls (group coded player = 1, control = 0), in the units of each parameter. Model 1, adjusted for age; Model 2, age, height and BMI; Model 3, age and circumference of the limb segment over which the response was recorded (calf, shoulder, upper-arm or forearm circumference). q, Benjamini–Hochberg FDR across all 110 NCS parameters within each model. Complete results for all parameters are given in Supplementary Table S1 (Model 1) and Supplementary Table S3 (Models 2 and 3).

**Table 2:** Lower-extremity nerve conduction parameters in American football players and controls.

Parameter	Players (*n* = 36)	Controls (*n* = 39)	Mean Difference (95% CI)	Hedges’ g (95% CI)	*p*	q
Right common peroneal nerve (motor)						
CMAP amplitude, ankle (mV)	4.79 ± 1.81	4.75 ± 1.48	0.04 (−0.72 to 0.81)	0.03 (−0.43 to 0.48)	0.907	0.959
CMAP amplitude, fibular head (mV)	4.07 ± 1.49	4.36 ± 1.42	−0.29 (−0.96 to 0.38)	−0.20 (−0.65 to 0.26)	0.397	0.692
CMAP amplitude, popliteal fossa (mV)	3.71 ± 1.35	4.03 ± 1.43	−0.32 (−0.96 to 0.32)	−0.23 (−0.68 to 0.23)	0.322	0.643
MCV, distal segment (m/s)	50.8 ± 5.3	47.7 ± 4.2	3.1 (0.9 to 5.3)	0.64 (0.18 to 1.11)	0.007	0.070
MCV, proximal segment (m/s)	58.2 ± 6.3	57.8 ± 5.4	0.4 (−2.3 to 3.1)	0.06 (−0.39 to 0.51)	0.792	0.907
Motor latency, ankle (ms)	3.76 ± 0.52	3.92 ± 0.62	−0.16 (−0.42 to 0.11)	−0.27 (−0.73 to 0.18)	0.238	0.568
Motor latency, fibular head (ms)	10.76 ± 0.99	11.34 ± 1.07	−0.58 (−1.06 to −0.11)	−0.56 (−1.02 to −0.10)	0.017	0.092
Motor latency, popliteal fossa (ms)	12.51 ± 0.99	12.87 ± 1.12	−0.37 (−0.85 to 0.12)	−0.34 (−0.80 to 0.12)	0.139	0.410
Right tibial nerve (motor)						
CMAP amplitude, ankle (mV)	8.44 ± 2.38	10.49 ± 3.37	−2.05 (−3.39 to −0.71)	−0.69 (−1.16 to −0.22)	0.003	0.043
CMAP amplitude, popliteal fossa (mV)	7.00 ± 1.97 (35)	8.97 ± 3.24	−1.96 (−3.19 to −0.73)	−0.72 (−1.19 to −0.25)	0.002	0.035
MCV (m/s)	47.9 ± 4.9	47.8 ± 3.8 (38)	0.1 (−1.9 to 2.2)	0.03 (−0.42 to 0.49)	0.892	0.959
F–M latency (ms)	45.29 ± 3.63	46.21 ± 3.91	−0.92 (−2.66 to 0.82)	−0.24 (−0.69 to 0.21)	0.296	0.612
Motor latency, ankle (ms)	3.58 ± 0.53	3.56 ± 0.56	0.02 (−0.24 to 0.27)	0.03 (−0.42 to 0.48)	0.901	0.959
Motor latency, popliteal fossa (ms)	12.57 ± 1.15	12.88 ± 1.28	−0.31 (−0.87 to 0.25)	−0.25 (−0.71 to 0.20)	0.275	0.606
Right sural nerve (sensory)						
SNAP amplitude (µV)	17.58 ± 6.10	21.88 ± 9.37	−4.29 (−7.91 to −0.67)	−0.53 (−0.99 to −0.07)	0.021	0.099
SCV (m/s)	55.4 ± 6.9	53.1 ± 7.7	2.4 (−1.0 to 5.7)	0.32 (−0.13 to 0.78)	0.163	0.438
Peak latency (ms)	3.09 ± 0.35	3.07 ± 0.38	0.03 (−0.14 to 0.19)	0.07 (−0.38 to 0.53)	0.748	0.884
Left common peroneal nerve (motor)						
CMAP amplitude, ankle (mV)	5.14 ± 1.60	4.88 ± 1.55	0.26 (−0.47 to 0.98)	0.16 (−0.29 to 0.62)	0.479	0.726
CMAP amplitude, fibular head (mV)	4.46 ± 1.42	4.42 ± 1.52	0.04 (−0.63 to 0.72)	0.03 (−0.42 to 0.48)	0.902	0.959
CMAP amplitude, popliteal fossa (mV)	4.21 ± 1.57	4.03 ± 1.59	0.18 (−0.55 to 0.90)	0.11 (−0.34 to 0.57)	0.627	0.797
MCV, distal segment (m/s)	50.5 ± 5.3	48.7 ± 4.7	1.7 (−0.6 to 4.1)	0.34 (−0.11 to 0.80)	0.141	0.410
MCV, proximal segment (m/s)	58.3 ± 5.6	55.9 ± 7.3	2.4 (−0.6 to 5.4)	0.36 (−0.10 to 0.82)	0.115	0.409
Motor latency, ankle (ms)	3.89 ± 0.64	3.98 ± 0.52	−0.09 (−0.36 to 0.18)	−0.16 (−0.61 to 0.30)	0.496	0.738
Motor latency, fibular head (ms)	10.73 ± 1.12	10.97 ± 1.06	−0.24 (−0.74 to 0.26)	−0.22 (−0.67 to 0.23)	0.340	0.646
Motor latency, popliteal fossa (ms)	12.47 ± 1.17	12.62 ± 1.23	−0.15 (−0.70 to 0.40)	−0.12 (−0.58 to 0.33)	0.587	0.788
Left tibial nerve (motor)						
CMAP amplitude, ankle (mV)	8.73 ± 2.52	10.61 ± 3.86	−1.88 (−3.37 to −0.39)	−0.57 (−1.03 to −0.10)	0.014	0.092
CMAP amplitude, popliteal fossa (mV)	7.00 ± 1.98	9.03 ± 3.77	−2.03 (−3.41 to −0.65)	−0.66 (−1.12 to −0.19)	0.005	0.056
MCV (m/s)	49.4 ± 4.5	47.6 ± 4.3 (38)	1.8 (−0.2 to 3.9)	0.40 (−0.06 to 0.86)	0.084	0.330
F–M latency (ms)	45.28 ± 3.51	46.31 ± 3.47	−1.03 (−2.64 to 0.58)	−0.29 (−0.75 to 0.16)	0.206	0.520
Motor latency, ankle (ms)	3.71 ± 0.65	3.81 ± 0.78	−0.10 (−0.43 to 0.23)	−0.14 (−0.60 to 0.31)	0.532	0.770
Motor latency, popliteal fossa (ms)	12.44 ± 1.22	13.16 ± 1.42	−0.72 (−1.33 to −0.12)	−0.54 (−1.00 to −0.08)	0.020	0.099
Left sural nerve (sensory)						
SNAP amplitude (µV)	17.33 ± 5.83	21.77 ± 8.85	−4.44 (−7.87 to −1.01)	−0.58 (−1.04 to −0.12)	0.012	0.087
SCV (m/s)	55.9 ± 7.6	51.3 ± 7.7	4.6 (1.0 to 8.1)	0.59 (0.13 to 1.05)	0.012	0.087
Peak latency (ms)	3.11 ± 0.46	3.16 ± 0.46	−0.04 (−0.26 to 0.17)	−0.09 (−0.55 to 0.36)	0.680	0.831

Data are mean ± SD; numbers in parentheses give n when lower than the full group because of missing or implausible values (Supplementary Table S2). Mean difference = players minus controls (Welch 95% CI); *p*, Welch’s *t*-test; q, Benjamini–Hochberg FDR across all 110 NCS comparisons in Table 2, Table 3 and Table 4. CMAP, compound muscle action potential; MCV, motor conduction velocity; SCV, sensory conduction velocity; SNAP, sensory nerve action potential.

**Table 3:** Shoulder-girdle and proximal upper-extremity nerve conduction parameters in American football players and controls.

Parameter	Players (*n* = 36)	Controls (*n* = 39)	Mean Difference (95% CI)	Hedges’ g (95% CI)	*p*	q
Right accessory nerve						
CMAP amplitude (upper trapezius) (mV)	7.43 ± 3.35	7.03 ± 2.56	0.40 (−0.98 to 1.79)	0.13 (−0.32 to 0.59)	0.562	0.773
Motor latency (ms)	3.70 ± 0.86 (35)	2.74 ± 0.85	0.96 (0.56 to 1.36)	1.11 (0.62 to 1.60)	<0.001	<0.001
Right axillary nerve						
CMAP amplitude (deltoid) (mV)	6.82 ± 3.28	7.31 ± 2.41	−0.50 (−1.84 to 0.84)	−0.17 (−0.63 to 0.28)	0.460	0.726
Motor latency (ms)	3.34 ± 1.07	2.81 ± 0.50	0.53 (0.14 to 0.92)	0.63 (0.17 to 1.10)	0.009	0.080
Right musculocutaneous nerve						
CMAP amplitude (biceps brachii) (mV)	7.41 ± 3.03	6.96 ± 2.20	0.44 (−0.79 to 1.67)	0.17 (−0.29 to 0.62)	0.477	0.726
Motor latency (ms)	2.80 ± 0.76	3.29 ± 0.93	−0.49 (−0.88 to −0.10)	−0.56 (−1.03 to −0.10)	0.015	0.092
Right medial antebrachial cutaneous nerve						
SNAP amplitude (µV)	12.31 ± 4.32	12.98 ± 3.56	−0.67 (−2.50 to 1.16)	−0.17 (−0.62 to 0.29)	0.469	0.726
SCV (m/s)	70.5 ± 6.8	68.9 ± 9.0 (38)	1.6 (−2.1 to 5.3)	0.20 (−0.26 to 0.65)	0.391	0.692
Peak latency (ms)	2.05 ± 0.37	2.00 ± 0.30	0.06 (−0.10 to 0.21)	0.17 (−0.28 to 0.63)	0.457	0.726
Right lateral antebrachial cutaneous nerve						
SNAP amplitude (µV)	21.23 ± 8.22	21.61 ± 6.25	−0.38 (−3.77 to 3.01)	−0.05 (−0.51 to 0.40)	0.822	0.923
SCV (m/s)	73.2 ± 6.7 (31)	74.2 ± 6.7 (38)	−1.0 (−4.2 to 2.3)	−0.14 (−0.62 to 0.33)	0.550	0.771
Peak latency (ms)	1.77 ± 0.36	1.78 ± 0.23	−0.01 (−0.15 to 0.13)	−0.02 (−0.48 to 0.43)	0.921	0.965
Left accessory nerve						
CMAP amplitude (upper trapezius) (mV)	6.82 ± 2.75	7.13 ± 2.47	−0.31 (−1.52 to 0.90)	−0.12 (−0.57 to 0.34)	0.608	0.789
Motor latency (ms)	3.63 ± 0.94	2.69 ± 0.75	0.94 (0.54 to 1.33)	1.09 (0.61 to 1.58)	<0.001	<0.001
Left axillary nerve						
CMAP amplitude (deltoid) (mV)	7.06 ± 2.63	7.25 ± 2.72	−0.19 (−1.42 to 1.04)	−0.07 (−0.52 to 0.38)	0.762	0.892
Motor latency (ms)	3.16 ± 0.70	3.00 ± 0.51	0.16 (−0.13 to 0.44)	0.26 (−0.20 to 0.71)	0.268	0.602
Left musculocutaneous nerve						
CMAP amplitude (biceps brachii) (mV)	7.03 ± 2.34	7.02 ± 2.45	0.02 (−1.08 to 1.12)	0.01 (−0.45 to 0.46)	0.974	0.983
Motor latency (ms)	2.74 ± 0.83	3.38 ± 0.88	−0.64 (−1.04 to −0.25)	−0.75 (−1.21 to −0.28)	0.002	0.031
Left medial antebrachial cutaneous nerve						
SNAP amplitude (µV)	14.09 ± 5.23	13.12 ± 3.13	0.97 (−1.04 to 2.99)	0.23 (−0.23 to 0.68)	0.337	0.646
SCV (m/s)	70.6 ± 4.5 (35)	70.5 ± 7.3 (38)	0.1 (−2.7 to 2.9)	0.01 (−0.44 to 0.47)	0.950	0.967
Peak latency (ms)	2.07 ± 0.26	2.07 ± 0.23	0.00 (−0.11 to 0.11)	0.00 (−0.45 to 0.46)	0.983	0.983
Left lateral antebrachial cutaneous nerve						
SNAP amplitude (µV)	21.78 ± 8.80	21.95 ± 6.94	−0.16 (−3.84 to 3.51)	−0.02 (−0.47 to 0.43)	0.930	0.965
SCV (m/s)	74.4 ± 6.6 (31)	73.5 ± 8.5 (38)	0.9 (−2.8 to 4.5)	0.11 (−0.36 to 0.59)	0.638	0.797
Peak latency (ms)	1.84 ± 0.27	1.88 ± 0.29	−0.04 (−0.17 to 0.09)	−0.14 (−0.59 to 0.32)	0.554	0.771

Data are mean ± SD; numbers in parentheses give n when lower than the full group because of missing or implausible values (Supplementary Table S2). Mean difference = players minus controls (Welch 95% CI); *p*, Welch’s *t*-test; q, Benjamini–Hochberg FDR across all 110 NCS comparisons in Table 2, Table 3 and Table 4. CMAP, compound muscle action potential; MCV, motor conduction velocity; SCV, sensory conduction velocity; SNAP, sensory nerve action potential.

**Table 4:** Median, ulnar and radial nerve conduction parameters in American football players and controls.

Parameter	Players (*n* = 36)	Controls (*n* = 39)	Mean Difference (95% CI)	Hedges’ g (95% CI)	*p*	q
Right median nerve						
SNAP amplitude (2nd digit) (µV)	41.80 ± 13.71	52.77 ± 12.09	−10.97 (−16.94 to −5.00)	−0.84 (−1.32 to −0.37)	<0.001	0.011
SCV (m/s)	64.7 ± 8.4	65.6 ± 6.5	−0.9 (−4.4 to 2.6)	−0.12 (−0.58 to 0.33)	0.594	0.788
Sensory onset latency (ms)	3.01 ± 0.34	2.97 ± 0.33	0.04 (−0.11 to 0.20)	0.12 (−0.33 to 0.58)	0.592	0.788
CMAP amplitude, wrist (mV)	9.79 ± 2.73	10.52 ± 3.64	−0.72 (−2.20 to 0.75)	−0.22 (−0.68 to 0.23)	0.331	0.646
CMAP amplitude, elbow (mV)	8.96 ± 2.86	10.10 ± 3.40	−1.14 (−2.59 to 0.30)	−0.36 (−0.82 to 0.10)	0.119	0.409
MCV (m/s)	58.6 ± 4.7	59.4 ± 3.8 (38)	−0.9 (−2.9 to 1.1)	−0.20 (−0.66 to 0.25)	0.385	0.692
Motor latency, wrist (ms)	3.06 ± 0.40	3.16 ± 0.36	−0.11 (−0.28 to 0.07)	−0.28 (−0.73 to 0.18)	0.232	0.567
Motor latency, elbow (ms)	7.37 ± 0.52	7.46 ± 0.66 (38)	−0.10 (−0.37 to 0.18)	−0.16 (−0.62 to 0.29)	0.482	0.726
Right ulnar nerve						
SNAP amplitude (5th digit) (µV)	32.53 ± 11.67	44.30 ± 13.09	−11.77 (−17.47 to −6.07)	−0.94 (−1.41 to −0.46)	<0.001	0.004
SCV (m/s)	63.8 ± 6.3 (34)	61.8 ± 5.5	1.9 (−0.8 to 4.7)	0.33 (−0.14 to 0.79)	0.168	0.441
Sensory onset latency (ms)	2.61 ± 0.27	2.72 ± 0.42	−0.10 (−0.27 to 0.06)	−0.29 (−0.74 to 0.17)	0.208	0.520
CMAP amplitude, wrist (mV)	8.48 ± 1.71	9.00 ± 1.31 (35)	−0.52 (−1.24 to 0.20)	−0.34 (−0.81 to 0.13)	0.154	0.423
CMAP amplitude, below elbow (mV)	8.07 ± 1.60	8.28 ± 1.32 (35)	−0.21 (−0.90 to 0.49)	−0.14 (−0.61 to 0.33)	0.552	0.771
CMAP amplitude, above elbow (mV)	7.45 ± 1.47	7.80 ± 1.36 (35)	−0.35 (−1.02 to 0.32)	−0.24 (−0.71 to 0.22)	0.300	0.612
MCV (m/s)	63.7 ± 7.6	61.3 ± 5.4 (35)	2.4 (−0.7 to 5.5)	0.36 (−0.11 to 0.83)	0.131	0.410
Motor latency, wrist (ms)	2.47 ± 0.44	2.60 ± 0.58 (35)	−0.13 (−0.37 to 0.11)	−0.25 (−0.72 to 0.22)	0.293	0.612
Motor latency, below elbow (ms)	6.07 ± 0.64	6.56 ± 0.84 (35)	−0.50 (−0.86 to −0.14)	−0.66 (−1.14 to −0.18)	0.007	0.070
Motor latency, above elbow (ms)	7.69 ± 0.76	8.24 ± 0.91 (35)	−0.55 (−0.94 to −0.15)	−0.64 (−1.12 to −0.17)	0.008	0.074
Right radial nerve						
SNAP amplitude (1st digit) (µV)	25.87 ± 9.75	29.09 ± 7.26 (38)	−3.21 (−7.22 to 0.79)	−0.37 (−0.83 to 0.09)	0.114	0.409
SCV (m/s)	67.5 ± 10.0 (35)	66.5 ± 7.6 (38)	1.0 (−3.2 to 5.2)	0.11 (−0.35 to 0.57)	0.634	0.797
Sensory onset latency (ms)	1.92 ± 0.34	2.03 ± 0.30 (38)	−0.11 (−0.26 to 0.04)	−0.34 (−0.80 to 0.12)	0.145	0.410
CMAP amplitude, forearm (mV)	5.70 ± 1.62	6.03 ± 2.16 (38)	−0.33 (−1.21 to 0.56)	−0.17 (−0.63 to 0.29)	0.463	0.726
CMAP amplitude, elbow (mV)	4.94 ± 1.56	4.58 ± 1.06 (38)	0.36 (−0.26 to 0.98)	0.27 (−0.19 to 0.73)	0.252	0.580
MCV (m/s)	63.4 ± 10.3	60.3 ± 7.1 (38)	3.0 (−1.1 to 7.2)	0.34 (−0.12 to 0.80)	0.146	0.410
Motor latency, forearm (ms)	2.09 ± 0.43	2.14 ± 0.72 (38)	−0.04 (−0.32 to 0.23)	−0.07 (−0.53 to 0.38)	0.745	0.884
Motor latency, elbow (ms)	5.63 ± 0.61	5.54 ± 1.56 (38)	0.09 (−0.46 to 0.64)	0.07 (−0.38 to 0.53)	0.742	0.884
Left median nerve						
SNAP amplitude (2nd digit) (µV)	43.29 ± 15.69	50.62 ± 12.97	−7.33 (−13.99 to −0.67)	−0.51 (−0.97 to −0.05)	0.032	0.139
SCV (m/s)	63.6 ± 9.2	62.4 ± 6.0	1.2 (−2.4 to 4.8)	0.15 (−0.30 to 0.61)	0.508	0.745
Sensory onset latency (ms)	3.13 ± 0.38	3.11 ± 0.30	0.02 (−0.14 to 0.18)	0.06 (−0.39 to 0.52)	0.786	0.907
CMAP amplitude, wrist (mV)	8.92 ± 2.16	10.58 ± 3.59	−1.66 (−3.02 to −0.31)	−0.55 (−1.01 to −0.09)	0.017	0.092
CMAP amplitude, elbow (mV)	8.43 ± 2.00	10.00 ± 3.31	−1.57 (−2.83 to −0.32)	−0.56 (−1.03 to −0.10)	0.015	0.092
MCV (m/s)	58.9 ± 4.9	59.9 ± 4.7	−1.0 (−3.2 to 1.2)	−0.21 (−0.66 to 0.24)	0.362	0.675
Motor latency, wrist (ms)	3.19 ± 0.43	3.21 ± 0.34	−0.01 (−0.19 to 0.16)	−0.04 (−0.49 to 0.42)	0.875	0.959
Motor latency, elbow (ms)	7.38 ± 0.60	7.50 ± 0.79	−0.12 (−0.44 to 0.20)	−0.16 (−0.62 to 0.29)	0.473	0.726
Left ulnar nerve						
SNAP amplitude (5th digit) (µV)	32.46 ± 12.48	42.77 ± 11.67	−10.31 (−15.88 to −4.73)	−0.85 (−1.32 to −0.37)	<0.001	0.011
SCV (m/s)	61.7 ± 5.5 (35)	63.1 ± 5.8	−1.4 (−4.1 to 1.2)	−0.25 (−0.71 to 0.21)	0.284	0.612
Sensory onset latency (ms)	2.66 ± 0.32	2.64 ± 0.34	0.02 (−0.13 to 0.17)	0.05 (−0.40 to 0.51)	0.816	0.923
CMAP amplitude, wrist (mV)	8.82 ± 1.81	9.25 ± 2.38	−0.44 (−1.40 to 0.53)	−0.20 (−0.66 to 0.25)	0.374	0.685
CMAP amplitude, below elbow (mV)	7.98 ± 1.67	8.69 ± 2.36	−0.70 (−1.64 to 0.23)	−0.34 (−0.79 to 0.12)	0.139	0.410
CMAP amplitude, above elbow (mV)	7.67 ± 1.61	8.38 ± 2.30	−0.71 (−1.62 to 0.20)	−0.35 (−0.81 to 0.10)	0.123	0.410
MCV (m/s)	63.5 ± 7.2	62.2 ± 7.4	1.3 (−2.1 to 4.6)	0.17 (−0.28 to 0.63)	0.455	0.726
Motor latency, wrist (ms)	2.51 ± 0.36	2.70 ± 0.50	−0.19 (−0.39 to 0.01)	−0.43 (−0.89 to 0.03)	0.062	0.253
Motor latency, below elbow (ms)	6.17 ± 0.59	6.60 ± 1.04	−0.43 (−0.81 to −0.04)	−0.50 (−0.96 to −0.04)	0.030	0.139
Motor latency, above elbow (ms)	7.87 ± 0.73	8.36 ± 1.01	−0.49 (−0.89 to −0.09)	−0.55 (−1.01 to −0.08)	0.018	0.096
Left radial nerve						
SNAP amplitude (1st digit) (µV)	25.96 ± 8.18	26.83 ± 8.32 (38)	−0.88 (−4.70 to 2.95)	−0.11 (−0.56 to 0.35)	0.649	0.802
SCV (m/s)	65.7 ± 8.0	65.4 ± 6.8 (38)	0.2 (−3.2 to 3.7)	0.03 (−0.42 to 0.49)	0.893	0.959
Sensory onset latency (ms)	2.00 ± 0.30	2.12 ± 0.33 (38)	−0.12 (−0.27 to 0.02)	−0.39 (−0.85 to 0.07)	0.094	0.357
CMAP amplitude, forearm (mV)	5.33 ± 1.35	5.55 ± 1.16	−0.22 (−0.80 to 0.37)	−0.17 (−0.62 to 0.28)	0.463	0.726
CMAP amplitude, elbow (mV)	4.71 ± 1.44	4.73 ± 1.10	−0.02 (−0.61 to 0.57)	−0.02 (−0.47 to 0.44)	0.946	0.967
MCV (m/s)	65.9 ± 8.2	66.8 ± 7.9	−1.0 (−4.7 to 2.7)	−0.12 (−0.57 to 0.34)	0.610	0.789
Motor latency, forearm (ms)	2.12 ± 0.59	2.25 ± 0.37	−0.13 (−0.36 to 0.10)	−0.27 (−0.72 to 0.19)	0.253	0.580
Motor latency, elbow (ms)	5.39 ± 0.72	5.10 ± 0.54	0.29 (0.00 to 0.59)	0.46 (0.00 to 0.91)	0.053	0.226

Data are mean ± SD; numbers in parentheses give n when lower than the full group because of missing or implausible values (Supplementary Table S2). Mean difference = players minus controls (Welch 95% CI); *p*, Welch’s *t*-test; q, Benjamini–Hochberg FDR across all 110 NCS comparisons in Table 2, Table 3 and Table 4. CMAP, compound muscle action potential; MCV, motor conduction velocity; SCV, sensory conduction velocity; SNAP, sensory nerve action potential.

**Table 5:** Demographic and anthropometric characteristics of American football players and controls.

Parameter	Players (*n* = 36)	Controls (*n* = 39)	Mean Difference (95% CI)	Hedges’ g (95% CI)	*p*	q
Age (years)	22.2 ± 2.8	20.1 ± 1.5	2.2 (1.1 to 3.3)	0.98 (0.50 to 1.46)	<0.001	–
Height (m)	1.81 ± 0.07	1.80 ± 0.07	0.01 (−0.02 to 0.04)	0.17 (−0.28 to 0.63)	0.455	0.493
Body weight (kg)	88.2 ± 17.8	80.5 ± 14.2	7.7 (0.3 to 15.2)	0.48 (0.02 to 0.94)	0.043	0.111
Body mass index (kg/m^2^)	26.8 ± 4.8	24.8 ± 4.0	2.0 (−0.1 to 4.0)	0.44 (−0.02 to 0.90)	0.059	0.118
Shoulder circumference (cm)	53.7 ± 5.5	42.8 ± 3.1	11.0 (8.9 to 13.1)	2.44 (1.84 to 3.04)	<0.001	<0.001
Upper arm length (cm)	34.2 ± 2.4	34.9 ± 3.5	−0.7 (−2.0 to 0.7)	−0.23 (−0.68 to 0.23)	0.322	0.380
Forearm length (cm)	27.8 ± 2.7	28.4 ± 2.6	−0.6 (−1.9 to 0.6)	−0.24 (−0.69 to 0.22)	0.305	0.380
Upper arm circumference (cm)	33.8 ± 2.8	30.8 ± 4.5	3.0 (1.3 to 4.7)	0.78 (0.31 to 1.25)	<0.001	0.004
Forearm circumference (cm)	28.9 ± 2.2	26.3 ± 3.1	2.6 (1.3 to 3.8)	0.93 (0.45 to 1.41)	<0.001	<0.001
Total lower-extremity length (cm)	104.9 ± 5.5	106.6 ± 5.9	−1.7 (−4.3 to 0.9)	−0.30 (−0.76 to 0.16)	0.193	0.312
Thigh length (cm)	53.5 ± 3.6	53.7 ± 3.9	−0.2 (−2.0 to 1.5)	−0.06 (−0.51 to 0.40)	0.801	0.801
Lower leg length (cm)	52.7 ± 4.4	50.5 ± 3.7	2.2 (0.3 to 4.1)	0.53 (0.07 to 1.00)	0.023	0.076
Thigh circumference (cm)	57.1 ± 6.6	54.2 ± 6.8	2.9 (−0.2 to 6.0)	0.43 (−0.03 to 0.89)	0.064	0.118
Calf circumference (cm)	40.7 ± 3.9	39.5 ± 4.3	1.2 (−0.7 to 3.1)	0.28 (−0.17 to 0.74)	0.216	0.312

Data are mean ± SD. Mean difference = players minus controls (Welch 95% CI); *p*, Welch’s *t*-test; q, Benjamini–Hochberg FDR across the 12 anthropometric variables (total upper extremity length not included). Limb measurements refer to the right side (see Section 2.3). Total upper extremity length was excluded because of inconsistent landmark use (see Section 2.3).

## Data Availability

The original contributions presented in this study are included in the article and its Supplementary Materials. The de-identified dataset and the analysis code are available from the corresponding author upon reasonable request.

## Supplementary Materials

The following supporting information can be downloaded at: https://www.mdpi.com/article/10.3390/diagnostics16203343/s1, Table S1. Nerve conduction parameters: medians (interquartile range), Mann–Whitney U test, and age-adjusted linear models (Model 1) for all 110 parameters; Table S2. NCS values that could not be verified against the original EMG reports, and their handling in the main analysis (22 values from 9 participants); Table S3. Adjusted differences between football players and controls for all 110 NCS parameters: Model 2 (age, height and BMI) and Model 3 (age and local limb circumference); Table S4. Sensitivity of the age-adjusted results (Model 1) to the handling of values that could not be verified against the EMG reports.

## Abbreviations

The following abbreviations are used in this manuscript:

AAEM	American Association of Electrodiagnostic Medicine
BMI	Body mass index
CI	Confidence interval
CMAP	Compound muscle action potential
EMG	Electromyography
F-M	F-wave motor latency
FDR	False discovery rate
ISAK	International Society for the Advancement of Kinanthropometry
MCV	Motor conduction velocity
NCS	Nerve conduction study
NFL	National Football League
SCV	Sensory conduction velocity
SD	Standard deviation
SNAP	Sensory nerve action potential
