# Supplementary material for: Peripheral Nerve Conduction and Anthropometric Characteristics in Elite American Football Players: An Exploratory Cross-Sectional Study
Source: Diagnostics (Basel). 2026 Oct 14;16(20):3343. doi: 10.3390/diagnostics16203343 (PMC13703672; doi:10.3390/diagnostics16203343)
Supplement: Supplementary file 1 [file diagnostics-16-03343-s001.zip › diagnostics-4578243-supplementary.pdf]

Supplementary Materials

Peripheral Nerve Conduction and Anthropometric Characteristics in Elite American Football Players: An Exploratory Cross-Sectional Study

Table S1. Nerve conduction parameters: medians (interquartile range), Mann–Whitney U test, and age-adjusted linear models (Model 1) for all 110 parameters.

Parameter	Football players, median (IQR)	Controls, median (IQR)	p (Mann–Whitney)	Model 1 β (95% CI)	p	q
Right common peroneal nerve (motor)						
CMAP amplitude, ankle (mV)	4.45 (3.65–5.40)	4.60 (3.85–5.70)	0.746	0.36 (−0.48 to 1.20)	0.393	0.682
CMAP amplitude, fibular head (mV)	3.85 (3.20–4.75)	4.20 (3.30–5.00)	0.321	0.04 (−0.69 to 0.77)	0.915	0.974
CMAP amplitude, popliteal fossa (mV)	3.60 (2.68–4.40)	4.00 (3.05–4.90)	0.340	−0.14 (−0.86 to 0.58)	0.702	0.898
MCV, distal segment (m/s)	50.5 (47.5–53.5)	47.6 (43.9–51.0)	0.015	3.6 (1.1 to 6.1)	0.005	0.087
MCV, proximal segment (m/s)	58.2 (54.4–63.2)	56.7 (53.5–62.5)	0.691	0.3 (−2.7 to 3.3)	0.841	0.946
Motor latency, ankle (ms)	3.81 (3.48–4.03)	3.93 (3.42–4.22)	0.379	−0.19 (−0.49 to 0.11)	0.202	0.494
Motor latency, fibular head (ms)	10.65 (10.17–11.12)	11.30 (10.65–12.15)	0.010	−0.69 (−1.22 to −0.16)	0.012	0.116
Motor latency, popliteal fossa (ms)	12.30 (11.80–13.10)	12.70 (12.25–13.70)	0.137	−0.47 (−1.02 to 0.08)	0.091	0.340
Right tibial nerve (motor)						
CMAP amplitude, ankle (mV)	8.35 (6.62–9.97)	9.90 (8.50–11.48)	0.008	−1.68 (−3.19 to −0.17)	0.030	0.182
CMAP amplitude, popliteal fossa (mV)	6.60 (5.70–8.15)	8.40 (6.60–10.80)	0.007	−1.73 (−3.14 to −0.32)	0.017	0.141
MCV (m/s)	48.1 (43.4–51.4)	47.0 (45.1–49.9)	0.978	0.6 (−1.6 to 2.9)	0.578	0.825
F–M latency (ms)	46.30 (42.88–47.82)	45.80 (43.70–49.20)	0.384	−0.88 (−2.85 to 1.08)	0.373	0.673
Motor latency, ankle (ms)	3.62 (3.18–3.95)	3.45 (3.25–3.97)	0.641	−0.03 (−0.32 to 0.25)	0.810	0.946
Motor latency, popliteal fossa (ms)	12.50 (11.97–13.10)	12.50 (12.05–13.70)	0.408	−0.41 (−1.04 to 0.22)	0.201	0.494
Right sural nerve (sensory)						
SNAP amplitude (μV)	16.65 (13.70–19.90)	17.80 (14.75–28.10)	0.059	−3.82 (−7.95 to 0.31)	0.069	0.305
SCV (m/s)	54.6 (50.7–59.5)	49.5 (47.2–58.8)	0.091	3.1 (−0.7 to 6.9)	0.108	0.362
Peak latency (ms)	2.99 (2.81–3.35)	3.11 (2.77–3.33)	0.820	−0.02 (−0.21 to 0.16)	0.807	0.946
Left common peroneal nerve (motor)						
CMAP amplitude, ankle (mV)	4.85 (4.05–6.22)	4.40 (3.65–6.20)	0.408	0.48 (−0.33 to 1.29)	0.238	0.535
CMAP amplitude, fibular head (mV)	4.40 (3.45–5.35)	3.90 (3.30–5.30)	0.746	0.20 (−0.56 to 0.96)	0.600	0.825
CMAP amplitude, popliteal fossa (mV)	4.05 (3.02–5.38)	3.60 (2.75–5.30)	0.517	0.26 (−0.55 to 1.08)	0.521	0.796
MCV, distal segment (m/s)	49.6 (46.1–54.7)	49.0 (44.0–52.1)	0.190	2.7 (0.2 to 5.3)	0.037	0.194
MCV, proximal segment (m/s)	58.1 (55.6–60.3)	56.3 (51.5–60.0)	0.178	2.9 (−0.5 to 6.3)	0.094	0.340
Motor latency, ankle (ms)	3.75 (3.46–4.34)	4.02 (3.66–4.20)	0.379	−0.13 (−0.43 to 0.17)	0.380	0.674
Motor latency, fibular head (ms)	10.65 (10.17–11.00)	10.80 (10.10–11.90)	0.329	−0.41 (−0.97 to 0.14)	0.142	0.412
Motor latency, popliteal fossa (ms)	12.35 (11.75–13.03)	12.50 (11.70–13.85)	0.667	−0.34 (−0.96 to 0.27)	0.273	0.557

Parameter	Football players, median (IQR)	Controls, median (IQR)	p (Mann–Whitney)	Model 1 β (95% CI)	p	q
Left tibial nerve (motor)						
CMAP amplitude, ankle (mV)	8.95 (6.85–9.95)	10.20 (8.20–12.60)	0.054	−1.43 (−3.12 to 0.26)	0.096	0.340
CMAP amplitude, popliteal fossa (mV)	6.95 (5.70–8.07)	8.10 (6.75–10.30)	0.013	−1.68 (−3.25 to −0.11)	0.037	0.194
MCV (m/s)	48.7 (46.1–53.0)	47.0 (44.4–50.9)	0.064	2.3 (0.1 to 4.6)	0.044	0.215
F–M latency (ms)	45.35 (43.83–47.73)	45.60 (43.95–48.65)	0.399	−1.06 (−2.87 to 0.76)	0.250	0.538
Motor latency, ankle (ms)	3.57 (3.36–3.86)	3.60 (3.25–4.23)	0.614	−0.16 (−0.53 to 0.22)	0.403	0.682
Motor latency, popliteal fossa (ms)	12.30 (11.88–13.05)	13.10 (12.40–13.90)	0.016	−0.81 (−1.50 to −0.12)	0.021	0.162
Left sural nerve (sensory)						
SNAP amplitude (μV)	17.00 (12.20–19.45)	18.80 (14.50–27.80)	0.032	−4.55 (−8.47 to −0.63)	0.024	0.162
SCV (m/s)	57.0 (50.1–62.1)	49.1 (46.0–55.8)	0.010	6.9 (3.2 to 10.7)	<0.001	0.012
Peak latency (ms)	3.00 (2.90–3.32)	3.17 (2.84–3.37)	0.402	−0.09 (−0.33 to 0.15)	0.447	0.744
Right accessory nerve						
CMAP amplitude (upper trapezius) (mV)	6.35 (4.80–9.93)	6.40 (5.30–8.30)	0.779	0.90 (−0.62 to 2.42)	0.240	0.535
Motor latency (ms)	3.90 (3.04–4.33)	2.56 (2.16–3.37)	<0.001	1.17 (0.74 to 1.60)	<0.001	<0.001
Right axillary nerve						
CMAP amplitude (deltoid) (mV)	6.05 (4.83–7.88)	6.90 (5.85–8.50)	0.129	0.06 (−1.40 to 1.52)	0.937	0.974
Motor latency (ms)	3.09 (2.71–3.55)	2.77 (2.56–3.17)	0.014	0.59 (0.16 to 1.02)	0.008	0.087
Right musculocutaneous nerve						
CMAP amplitude (biceps brachii) (mV)	6.55 (5.07–9.60)	6.70 (5.40–8.20)	0.865	1.06 (−0.27 to 2.39)	0.116	0.365
Motor latency (ms)	2.57 (2.30–3.08)	3.69 (2.40–4.00)	0.027	−0.62 (−1.05 to −0.18)	0.006	0.087
Right medial antebrachial cutaneous nerve						
SNAP amplitude (μV)	11.65 (9.67–14.00)	12.30 (10.25–15.40)	0.289	−1.20 (−3.22 to 0.83)	0.243	0.535
SCV (m/s)	71.6 (64.2–74.3)	70.0 (62.7–74.5)	0.436	2.3 (−1.8 to 6.5)	0.270	0.557
Peak latency (ms)	2.00 (1.84–2.21)	1.94 (1.79–2.16)	0.560	0.00 (−0.17 to 0.17)	0.978	0.982
Right lateral antebrachial cutaneous nerve						
SNAP amplitude (μV)	19.85 (14.15–27.23)	21.20 (16.90–24.65)	0.553	−0.05 (−3.82 to 3.72)	0.978	0.982
SCV (m/s)	72.3 (68.7–77.2)	73.8 (69.9–79.5)	0.369	−0.1 (−3.7 to 3.5)	0.940	0.974
Peak latency (ms)	1.69 (1.57–1.92)	1.79 (1.58–1.96)	0.381	−0.03 (−0.19 to 0.13)	0.695	0.898
Left accessory nerve						
CMAP amplitude (upper trapezius) (mV)	5.80 (4.62–8.12)	6.50 (5.25–8.80)	0.370	−0.23 (−1.59 to 1.12)	0.731	0.907
Motor latency (ms)	3.95 (2.81–4.27)	2.56 (2.13–3.17)	<0.001	1.29 (0.89 to 1.69)	<0.001	<0.001
Left axillary nerve						
CMAP amplitude (deltoid) (mV)	6.40 (5.15–8.62)	6.50 (5.55–9.10)	0.637	0.19 (−1.19 to 1.56)	0.786	0.940
Motor latency (ms)	2.92 (2.69–3.62)	2.96 (2.62–3.46)	0.521	0.15 (−0.17 to 0.46)	0.359	0.668
Left musculocutaneous nerve						

Parameter	Football players, median (IQR)	Controls, median (IQR)	p (Mann–Whitney)	Model 1 β (95% CI)	p	q
CMAP amplitude (biceps brachii) (mV)	6.70 (5.25–8.55)	7.00 (4.85–9.00)	0.979	0.20 (−1.04 to 1.44)	0.746	0.912
Motor latency (ms)	2.58 (2.23–2.86)	3.65 (2.70–3.99)	0.002	−0.82 (−1.25 to −0.38)	<0.001	0.012
Left medial antebrachial cutaneous nerve						
SNAP amplitude (μV)	12.60 (11.00–15.65)	12.90 (10.80–14.40)	0.710	1.05 (−1.16 to 3.27)	0.346	0.668
SCV (m/s)	71.4 (67.6–73.2)	70.8 (66.7–75.6)	0.965	−0.4 (−3.6 to 2.8)	0.822	0.946
Peak latency (ms)	2.08 (1.93–2.27)	2.06 (1.92–2.17)	0.762	−0.02 (−0.15 to 0.10)	0.720	0.907
Left lateral antebrachial cutaneous nerve						
SNAP amplitude (μV)	18.60 (15.17–30.02)	22.90 (18.30–26.30)	0.379	0.41 (−3.68 to 4.49)	0.843	0.946
SCV (m/s)	73.0 (69.7–77.8)	73.5 (66.9–78.5)	0.651	0.6 (−3.6 to 4.8)	0.779	0.940
Peak latency (ms)	1.86 (1.69–1.96)	1.83 (1.69–2.03)	0.911	−0.05 (−0.19 to 0.10)	0.504	0.791
Right median nerve						
SNAP amplitude (2nd digit) (μV)	41.90 (36.25–51.25)	53.50 (43.20–59.40)	0.001	−8.17 (−14.70 to −1.64)	0.015	0.137
SCV (m/s)	65.9 (59.3–70.5)	64.6 (61.4–69.0)	0.966	0.4 (−3.4 to 4.2)	0.831	0.946
Sensory onset latency (ms)	2.92 (2.79–3.17)	2.88 (2.75–3.12)	0.490	0.01 (−0.16 to 0.18)	0.898	0.974
CMAP amplitude, wrist (mV)	8.95 (7.95–10.97)	9.60 (8.00–13.40)	0.578	−0.49 (−2.16 to 1.19)	0.566	0.825
CMAP amplitude, elbow (mV)	7.85 (6.97–10.55)	9.40 (7.60–13.00)	0.132	−0.80 (−2.43 to 0.83)	0.331	0.662
MCV (m/s)	57.6 (54.3–61.0)	59.8 (56.5–61.5)	0.230	0.0 (−2.2 to 2.2)	0.982	0.982
Motor latency, wrist (ms)	2.98 (2.79–3.31)	3.10 (2.87–3.49)	0.197	−0.12 (−0.32 to 0.08)	0.224	0.524
Motor latency, elbow (ms)	7.24 (7.04–7.59)	7.37 (7.01–8.04)	0.538	−0.14 (−0.45 to 0.17)	0.364	0.668
Right ulnar nerve						
SNAP amplitude (5th digit) (μV)	32.10 (24.32–39.23)	44.30 (31.85–51.90)	<0.001	−9.40 (−15.74 to −3.07)	0.004	0.087
SCV (m/s)	62.9 (58.7–68.5)	61.5 (57.8–66.5)	0.220	2.6 (−0.5 to 5.7)	0.094	0.340
Sensory onset latency (ms)	2.60 (2.42–2.79)	2.58 (2.39–3.02)	0.518	−0.16 (−0.34 to 0.03)	0.090	0.340
CMAP amplitude, wrist (mV)	8.60 (7.20–9.80)	8.90 (8.60–9.75)	0.282	−0.24 (−1.03 to 0.56)	0.553	0.821
CMAP amplitude, below elbow (mV)	7.75 (7.20–9.43)	8.30 (7.45–9.15)	0.658	−0.13 (−0.91 to 0.65)	0.734	0.907
CMAP amplitude, above elbow (mV)	7.30 (6.78–8.60)	7.80 (6.85–8.95)	0.267	−0.19 (−0.94 to 0.55)	0.609	0.827
MCV (m/s)	63.2 (58.2–70.3)	62.3 (58.6–65.1)	0.223	4.0 (0.5 to 7.4)	0.024	0.162
Motor latency, wrist (ms)	2.33 (2.21–2.60)	2.42 (2.24–2.79)	0.407	−0.15 (−0.43 to 0.12)	0.265	0.557
Motor latency, below elbow (ms)	6.06 (5.51–6.40)	6.38 (5.92–7.03)	0.015	−0.54 (−0.94 to −0.15)	0.008	0.087
Motor latency, above elbow (ms)	7.60 (7.06–8.27)	8.08 (7.56–8.86)	0.020	−0.62 (−1.07 to −0.18)	0.006	0.087
Right radial nerve						
SNAP amplitude (1st digit) (μV)	25.15 (20.60–31.12)	28.35 (24.23–34.15)	0.051	−2.92 (−7.37 to 1.53)	0.195	0.494
SCV (m/s)	66.7 (60.8–70.7)	66.1 (61.7–71.6)	0.921	3.4 (−1.0 to 7.8)	0.131	0.389
Sensory onset latency (ms)	1.89 (1.75–2.07)	2.03 (1.80–2.14)	0.150	−0.15 (−0.31 to 0.02)	0.084	0.340
CMAP amplitude, forearm (mV)	5.35 (4.75–6.60)	5.50 (4.62–6.75)	0.600	−0.03 (−1.02 to 0.95)	0.948	0.974

Parameter	Football players, median (IQR)	Controls, median (IQR)	p (Mann–Whitney)	Model 1 β (95% CI)	p	q
CMAP amplitude, elbow (mV)	4.55 (3.85–5.90)	4.50 (3.92–5.28)	0.492	0.45 (−0.24 to 1.14)	0.198	0.494
MCV (m/s)	63.0 (55.5–68.3)	58.5 (55.4–62.9)	0.152	2.1 (−2.4 to 6.7)	0.353	0.668
Motor latency, forearm (ms)	2.09 (1.80–2.40)	1.94 (1.73–2.26)	0.665	0.06 (−0.24 to 0.37)	0.677	0.886
Motor latency, elbow (ms)	5.62 (5.16–6.02)	5.26 (4.92–5.48)	0.007	0.28 (−0.33 to 0.90)	0.358	0.668
Left median nerve						
SNAP amplitude (2nd digit) (μV)	41.65 (34.62–55.45)	48.00 (42.50–57.70)	0.075	−2.43 (−9.42 to 4.56)	0.490	0.781
SCV (m/s)	64.8 (58.4–69.1)	62.2 (58.9–67.7)	0.468	2.7 (−1.2 to 6.6)	0.177	0.488
Sensory onset latency (ms)	3.06 (2.91–3.26)	3.04 (2.92–3.26)	0.916	−0.01 (−0.19 to 0.17)	0.910	0.974
CMAP amplitude, wrist (mV)	8.85 (7.15–10.43)	9.50 (8.25–12.85)	0.089	−1.21 (−2.75 to 0.32)	0.119	0.365
CMAP amplitude, elbow (mV)	8.30 (6.83–9.90)	8.80 (7.75–12.25)	0.105	−1.12 (−2.53 to 0.30)	0.120	0.365
MCV (m/s)	57.5 (54.4–62.2)	59.3 (57.1–61.8)	0.301	−0.7 (−3.2 to 1.8)	0.575	0.825
Motor latency, wrist (ms)	3.14 (2.96–3.35)	3.21 (2.94–3.48)	0.726	−0.05 (−0.25 to 0.14)	0.590	0.825
Motor latency, elbow (ms)	7.27 (7.03–7.56)	7.35 (7.02–7.91)	0.603	−0.12 (−0.49 to 0.24)	0.511	0.792
Left ulnar nerve						
SNAP amplitude (5th digit) (μV)	31.60 (23.75–42.98)	42.20 (35.35–51.40)	0.001	−6.73 (−12.71 to −0.75)	0.028	0.181
SCV (m/s)	61.9 (57.5–65.6)	65.0 (57.8–67.2)	0.169	−1.1 (−4.0 to 1.9)	0.478	0.773
Sensory onset latency (ms)	2.58 (2.47–2.80)	2.58 (2.40–2.90)	0.660	−0.04 (−0.21 to 0.13)	0.651	0.874
CMAP amplitude, wrist (mV)	8.45 (7.38–10.60)	9.00 (7.30–10.45)	0.563	−0.09 (−1.18 to 1.00)	0.868	0.964
CMAP amplitude, below elbow (mV)	7.90 (6.95–8.80)	8.40 (7.00–9.60)	0.205	−0.40 (−1.45 to 0.66)	0.458	0.751
CMAP amplitude, above elbow (mV)	7.50 (6.55–8.53)	8.30 (6.45–9.25)	0.165	−0.43 (−1.46 to 0.59)	0.403	0.682
MCV (m/s)	63.0 (58.7–69.2)	61.7 (57.9–66.0)	0.340	3.1 (−0.6 to 6.8)	0.100	0.344
Motor latency, wrist (ms)	2.49 (2.31–2.62)	2.54 (2.33–3.07)	0.176	−0.15 (−0.38 to 0.08)	0.194	0.494
Motor latency, below elbow (ms)	6.12 (5.93–6.45)	6.58 (6.02–6.89)	0.025	−0.45 (−0.90 to −0.01)	0.045	0.215
Motor latency, above elbow (ms)	7.86 (7.34–8.28)	8.25 (7.61–8.75)	0.034	−0.49 (−0.96 to −0.03)	0.036	0.194
Left radial nerve						
SNAP amplitude (1st digit) (μV)	26.35 (20.46–31.07)	24.90 (20.22–32.30)	0.642	−0.91 (−5.20 to 3.38)	0.674	0.886
SCV (m/s)	65.2 (59.3–70.0)	64.3 (60.5–69.5)	0.987	1.0 (−2.8 to 4.8)	0.599	0.825
Sensory onset latency (ms)	1.96 (1.87–2.06)	2.04 (1.94–2.27)	0.043	−0.16 (−0.32 to 0.00)	0.054	0.248
CMAP amplitude, forearm (mV)	5.25 (4.30–6.30)	5.40 (4.50–6.50)	0.521	−0.21 (−0.86 to 0.45)	0.529	0.797
CMAP amplitude, elbow (mV)	4.55 (3.68–5.60)	4.60 (3.85–5.60)	0.803	−0.03 (−0.69 to 0.63)	0.930	0.974
MCV (m/s)	64.0 (60.3–72.4)	66.7 (62.0–72.8)	0.481	0.3 (−3.8 to 4.4)	0.884	0.973
Motor latency, forearm (ms)	2.09 (1.71–2.41)	2.24 (2.03–2.53)	0.154	−0.16 (−0.41 to 0.10)	0.219	0.524
Motor latency, elbow (ms)	5.54 (4.75–5.94)	5.09 (4.79–5.46)	0.068	0.24 (−0.09 to 0.56)	0.155	0.438

Model 1: linear regression with group (football player = 1, control = 0) and age; β = age-adjusted mean difference (players minus controls). q, Benjamini–Hochberg FDR across all 110 NCS parameters. IQR, interquartile range.

Table S2. NCS values that could not be verified against the original EMG reports, and their handling in the main analysis (22 values from 9 participants).

Participant	Parameter	Recorded value	Assessment	Main analysis
Player 4	Right ulnar nerve: SCV (m/s)	0	Rule 1: value ≤ 0 + Rule 2: velocity < 20 m/s + Rule 4 (implied distance 0.0 × pooled median)	**Set to missing**
Player 5	Right accessory nerve: Motor latency (ms)	31.7	Rule 3: 10.3 × pooled median	**Set to missing**
Player 5	Right ulnar nerve: SNAP amplitude (5th digit) (μV)	2.38	Markedly abnormal but no rule met; potentially genuine (0.06 × pooled median)	Retained
Player 5	Right ulnar nerve: SCV (m/s)	17.6	Rule 2: velocity < 20 m/s + Rule 4 (implied distance 0.3 × pooled median)	**Set to missing**
Player 5	Right ulnar nerve: Sensory onset latency (ms)	2.81	Within the observed range; no rule met (1.08 × pooled median)	Retained
Player 5	Left ulnar nerve: SNAP amplitude (5th digit) (μV)	2.63	Markedly abnormal but no rule met; potentially genuine (0.07 × pooled median)	Retained
Player 5	Left ulnar nerve: SCV (m/s)	17.2	Rule 2: velocity < 20 m/s + Rule 4 (implied distance 0.35 × pooled median)	**Set to missing**
Player 5	Left ulnar nerve: Sensory onset latency (ms)	3.33	Within the observed range; no rule met (1.29 × pooled median)	Retained
Player 7	Right radial nerve: SNAP amplitude (1st digit) (μV)	5.29	Markedly abnormal but no rule met; potentially genuine (0.19 × pooled median)	Retained
Player 7	Right radial nerve: SCV (m/s)	6.6	Rule 2: velocity < 20 m/s + Rule 4 (implied distance 0.11 × pooled median)	**Set to missing**
Player 7	Right radial nerve: Sensory onset latency (ms)	2.1	Within the observed range; no rule met (1.09 × pooled median)	Retained
Player 9	Right tibial nerve (motor): CMAP amplitude, popliteal fossa (mV)	54.2	Rule 3: 6.9 × pooled median	**Set to missing**
Player 17	Left medial antebrachial cutaneous nerve: SCV (m/s)	0.739	Rule 2: velocity < 20 m/s + Rule 4 (implied distance 0.01 × pooled median)	**Set to missing**
Player 23	Right lateral antebrachial cutaneous nerve: SCV (m/s)	0.717	Rule 2: velocity < 20 m/s + Rule 4 (implied distance 0.01 × pooled median)	**Set to missing**
Control c1	Right median nerve: MCV (m/s)	13	Rule 2: velocity < 20 m/s + Rule 4 (implied distance 0.17 × pooled median)	**Set to missing**
Control c5	Right median nerve: Motor latency, elbow (ms)	53.3	Rule 3: 7.3 × pooled median + Rule 4 (implied distance 10.7 × pooled median)	**Set to missing**
Control c5	Right radial nerve: CMAP amplitude, forearm (mV)	12.7	Markedly abnormal but no rule met; potentially genuine (2.33 × pooled median)	Retained
Control c5	Right radial nerve: Motor latency, elbow (ms)	11.2	Markedly abnormal but no rule met; potentially genuine (2.07 × pooled median)	Retained
Control c5	Right radial nerve: Motor latency, forearm (ms)	4.29	Markedly abnormal but no rule met; potentially genuine (2.18 × pooled median)	Retained

Participant	Parameter	Recorded value	Assessment	Main analysis
Control c7	Right radial nerve: CMAP amplitude, forearm (mV)	13.9	Markedly abnormal but no rule met; potentially genuine (2.55 × pooled median)	Retained
Control c7	Right radial nerve: Motor latency, elbow (ms)	12.3	Markedly abnormal but no rule met; potentially genuine (2.28 × pooled median)	Retained
Control c7	Right radial nerve: Motor latency, forearm (ms)	5.06	Markedly abnormal but no rule met; potentially genuine (2.57 × pooled median)	Retained

Rules (Section 2.5): (1) value ≤ 0; (2) conduction velocity < 20 or > 100 m/s; (3) latency or amplitude > 5 × the pooled median; (4) implied conduction distance (velocity × latency or latency difference) < 0.5 or > 2 × the pooled median. Rules were applied to all 8,250 NCS cells (8,196 recorded values) of the pooled dataset; every recording flagged by rule 4 contained a value meeting rules 1–3, and no other value met any rule. Values marked “Retained” were included in the main analysis and excluded in the stricter sensitivity analysis (Supplementary Table S4). In addition, 33 transcription errors were corrected and 13 empty fields were completed directly from the original EMG reports; these values were used in all analyses.

Table S3. Adjusted differences between football players and controls for all 110 NCS parameters: Model 2 (age, height and BMI) and Model 3 (age and local limb circumference).

Parameter	Model 2 β (95% CI)	p	q	Model 3 β (95% CI)	p	q
Right common peroneal nerve (motor) — Model 3 covariate: calf circumference						
CMAP amplitude, ankle (mV)	0.50 (−0.32 to 1.33)	0.229	0.514	0.41 (−0.43 to 1.24)	0.333	0.666
CMAP amplitude, fibular head (mV)	0.15 (−0.57 to 0.88)	0.673	0.896	0.07 (−0.66 to 0.81)	0.840	0.953
CMAP amplitude, popliteal fossa (mV)	−0.03 (−0.74 to 0.68)	0.930	0.956	−0.10 (−0.81 to 0.62)	0.785	0.928
MCV, distal segment (m/s)	4.0 (1.5 to 6.4)	0.002	0.044	3.6 (1.1 to 6.1)	0.006	0.127
MCV, proximal segment (m/s)	0.4 (−2.7 to 3.5)	0.802	0.923	0.0 (−2.9 to 3.0)	0.985	0.994
Motor latency, ankle (ms)	−0.22 (−0.52 to 0.08)	0.153	0.414	−0.21 (−0.51 to 0.09)	0.167	0.486
Motor latency, fibular head (ms)	−0.79 (−1.30 to −0.27)	0.004	0.065	−0.71 (−1.25 to −0.18)	0.009	0.148
Motor latency, popliteal fossa (ms)	−0.57 (−1.10 to −0.04)	0.035	0.195	−0.48 (−1.03 to 0.08)	0.091	0.372
Right tibial nerve (motor) — Model 3 covariate: calf circumference						
CMAP amplitude, ankle (mV)	−1.60 (−3.14 to −0.06)	0.042	0.209	−1.58 (−3.07 to −0.08)	0.039	0.227
CMAP amplitude, popliteal fossa (mV)	−1.61 (−3.04 to −0.18)	0.028	0.180	−1.65 (−3.05 to −0.24)	0.023	0.203
MCV (m/s)	0.9 (−1.4 to 3.2)	0.434	0.724	0.7 (−1.5 to 3.0)	0.520	0.824
F–M latency (ms)	−1.20 (−3.08 to 0.68)	0.207	0.495	−0.93 (−2.91 to 1.04)	0.350	0.675
Motor latency, ankle (ms)	−0.03 (−0.32 to 0.26)	0.835	0.923	−0.03 (−0.32 to 0.26)	0.836	0.953
Motor latency, popliteal fossa (ms)	−0.48 (−1.11 to 0.16)	0.138	0.410	−0.43 (−1.07 to 0.21)	0.182	0.488
Right sural nerve (sensory) — Model 3 covariate: calf circumference						
SNAP amplitude (μV)	−3.70 (−7.80 to 0.41)	0.077	0.283	−3.99 (−8.13 to 0.16)	0.059	0.296
SCV (m/s)	3.1 (−0.7 to 6.9)	0.105	0.340	3.0 (−0.8 to 6.8)	0.119	0.396
Peak latency (ms)	−0.02 (−0.20 to 0.17)	0.848	0.923	−0.01 (−0.20 to 0.17)	0.874	0.981
Left common peroneal nerve (motor) — Model 3 covariate: calf circumference						
CMAP amplitude, ankle (mV)	0.52 (−0.31 to 1.34)	0.216	0.496	0.48 (−0.34 to 1.30)	0.244	0.560
CMAP amplitude, fibular head (mV)	0.24 (−0.53 to 1.02)	0.534	0.827	0.19 (−0.58 to 0.96)	0.626	0.869
CMAP amplitude, popliteal fossa (mV)	0.31 (−0.52 to 1.15)	0.459	0.731	0.26 (−0.57 to 1.09)	0.532	0.824
MCV, distal segment (m/s)	3.0 (0.5 to 5.6)	0.020	0.149	2.7 (0.1 to 5.3)	0.039	0.227
MCV, proximal segment (m/s)	3.2 (−0.2 to 6.6)	0.062	0.255	2.7 (−0.7 to 6.0)	0.119	0.396
Motor latency, ankle (ms)	−0.14 (−0.45 to 0.16)	0.348	0.661	−0.13 (−0.43 to 0.17)	0.401	0.715
Motor latency, fibular head (ms)	−0.50 (−1.05 to 0.05)	0.073	0.275	−0.42 (−0.99 to 0.14)	0.137	0.432
Motor latency, popliteal fossa (ms)	−0.44 (−1.04 to 0.15)	0.144	0.414	−0.34 (−0.96 to 0.29)	0.284	0.601
Left tibial nerve (motor) — Model 3 covariate: calf circumference						
CMAP amplitude, ankle (mV)	−1.28 (−2.98 to 0.41)	0.136	0.410	−1.35 (−3.04 to 0.35)	0.117	0.396
CMAP amplitude, popliteal fossa (mV)	−1.52 (−3.09 to 0.06)	0.058	0.255	−1.60 (−3.17 to −0.03)	0.046	0.255
MCV (m/s)	2.7 (0.5 to 5.0)	0.019	0.149	2.4 (0.2 to 4.7)	0.037	0.227
F–M latency (ms)	−1.26 (−3.05 to 0.52)	0.162	0.418	−1.09 (−2.92 to 0.74)	0.238	0.557
Motor latency, ankle (ms)	−0.16 (−0.54 to 0.22)	0.414	0.712	−0.17 (−0.55 to 0.21)	0.371	0.691

Parameter	Model 2 β (95% CI)	p	q	Model 3 β (95% CI)	p	q
Motor latency, popliteal fossa (ms)	−0.89 (−1.58 to −0.20)	0.012	0.104	−0.84 (−1.53 to −0.15)	0.018	0.199
Left sural nerve (sensory) — Model 3 covariate: calf circumference						
SNAP amplitude (μV)	−4.20 (−8.09 to −0.30)	0.035	0.195	−4.53 (−8.49 to −0.56)	0.026	0.203
SCV (m/s)	7.0 (3.3 to 10.8)	<0.001	0.014	6.9 (3.1 to 10.7)	<0.001	0.028
Peak latency (ms)	−0.10 (−0.34 to 0.14)	0.398	0.712	−0.09 (−0.33 to 0.15)	0.469	0.795
Right accessory nerve — Model 3 covariate: shoulder circumference						
CMAP amplitude (upper trapezius) (mV)	0.89 (−0.65 to 2.43)	0.254	0.528	1.35 (−0.87 to 3.57)	0.228	0.545
Motor latency (ms)	1.12 (0.69 to 1.55)	<0.001	<0.001	1.01 (0.38 to 1.63)	0.002	0.077
Right axillary nerve — Model 3 covariate: upper arm circumference						
CMAP amplitude (deltoid) (mV)	0.40 (−0.98 to 1.77)	0.567	0.860	0.82 (−0.65 to 2.29)	0.269	0.592
Motor latency (ms)	0.56 (0.13 to 1.00)	0.011	0.104	0.49 (0.03 to 0.94)	0.036	0.227
Right musculocutaneous nerve — Model 3 covariate: upper arm circumference						
CMAP amplitude (biceps brachii) (mV)	1.14 (−0.21 to 2.50)	0.096	0.319	0.97 (−0.45 to 2.39)	0.178	0.488
Motor latency (ms)	−0.59 (−1.03 to −0.15)	0.009	0.104	−0.53 (−1.00 to −0.07)	0.026	0.203
Right medial antebrachial cutaneous nerve — Model 3 covariate: forearm circumference						
SNAP amplitude (μV)	−1.09 (−3.16 to 0.98)	0.296	0.582	−0.63 (−2.81 to 1.55)	0.564	0.828
SCV (m/s)	2.5 (−1.8 to 6.7)	0.246	0.520	3.1 (−1.4 to 7.6)	0.172	0.486
Peak latency (ms)	−0.02 (−0.19 to 0.15)	0.830	0.923	−0.05 (−0.23 to 0.13)	0.591	0.855
Right lateral antebrachial cutaneous nerve — Model 3 covariate: forearm circumference						
SNAP amplitude (μV)	0.58 (−3.12 to 4.28)	0.755	0.914	1.43 (−2.56 to 5.43)	0.477	0.795
SCV (m/s)	−0.5 (−4.2 to 3.1)	0.765	0.914	−0.6 (−4.5 to 3.3)	0.759	0.920
Peak latency (ms)	−0.05 (−0.21 to 0.10)	0.516	0.811	−0.10 (−0.27 to 0.06)	0.206	0.527
Left accessory nerve — Model 3 covariate: shoulder circumference						
CMAP amplitude (upper trapezius) (mV)	−0.23 (−1.62 to 1.15)	0.741	0.914	0.11 (−1.87 to 2.09)	0.915	0.987
Motor latency (ms)	1.23 (0.84 to 1.62)	<0.001	<0.001	1.21 (0.63 to 1.80)	<0.001	0.011
Left axillary nerve — Model 3 covariate: upper arm circumference						
CMAP amplitude (deltoid) (mV)	0.38 (−0.98 to 1.75)	0.578	0.860	0.64 (−0.80 to 2.08)	0.381	0.698
Motor latency (ms)	0.14 (−0.18 to 0.47)	0.381	0.709	0.11 (−0.23 to 0.44)	0.525	0.824
Left musculocutaneous nerve — Model 3 covariate: upper arm circumference						
CMAP amplitude (biceps brachii) (mV)	0.31 (−0.94 to 1.57)	0.619	0.871	0.39 (−0.93 to 1.71)	0.559	0.828
Motor latency (ms)	−0.79 (−1.23 to −0.36)	<0.001	0.015	−0.69 (−1.15 to −0.23)	0.004	0.097
Left medial antebrachial cutaneous nerve — Model 3 covariate: forearm circumference						
SNAP amplitude (μV)	1.20 (−1.04 to 3.43)	0.290	0.579	1.79 (−0.57 to 4.15)	0.134	0.432
SCV (m/s)	−0.6 (−3.9 to 2.6)	0.702	0.909	−0.7 (−4.2 to 2.8)	0.706	0.891
Peak latency (ms)	−0.03 (−0.16 to 0.10)	0.618	0.871	−0.06 (−0.19 to 0.08)	0.403	0.715
Left lateral antebrachial cutaneous nerve — Model 3 covariate: forearm circumference						

Parameter	Model 2 β (95% CI)	p	q	Model 3 β (95% CI)	p	q
SNAP amplitude (μV)	0.57 (−3.58 to 4.72)	0.785	0.919	1.36 (−3.03 to 5.76)	0.539	0.824
SCV (m/s)	0.5 (−3.8 to 4.8)	0.815	0.923	0.6 (−4.0 to 5.2)	0.791	0.928
Peak latency (ms)	−0.06 (−0.21 to 0.09)	0.441	0.724	−0.08 (−0.23 to 0.08)	0.339	0.666
Right median nerve — Model 3 covariate: forearm circumference						
SNAP amplitude (2nd digit) (μV)	−6.75 (−12.90 to −0.59)	0.032	0.195	−7.66 (−14.76 to −0.56)	0.035	0.227
SCV (m/s)	0.6 (−3.2 to 4.5)	0.742	0.914	−0.1 (−4.2 to 4.1)	0.974	0.994
Sensory onset latency (ms)	0.01 (−0.17 to 0.18)	0.941	0.958	0.00 (−0.19 to 0.19)	0.980	0.994
CMAP amplitude, wrist (mV)	−0.25 (−1.92 to 1.41)	0.762	0.914	0.03 (−1.76 to 1.83)	0.970	0.994
CMAP amplitude, elbow (mV)	−0.62 (−2.26 to 1.02)	0.451	0.730	−0.53 (−2.29 to 1.24)	0.552	0.828
MCV (m/s)	0.1 (−2.0 to 2.3)	0.893	0.935	0.1 (−2.3 to 2.5)	0.929	0.993
Motor latency, wrist (ms)	−0.11 (−0.32 to 0.09)	0.265	0.539	−0.07 (−0.29 to 0.14)	0.494	0.811
Motor latency, elbow (ms)	−0.16 (−0.47 to 0.15)	0.302	0.583	−0.06 (−0.40 to 0.28)	0.720	0.891
Right ulnar nerve — Model 3 covariate: forearm circumference						
SNAP amplitude (5th digit) (μV)	−8.30 (−14.50 to −2.09)	0.009	0.104	−8.53 (−15.40 to −1.65)	0.016	0.193
SCV (m/s)	2.7 (−0.5 to 5.8)	0.093	0.319	2.1 (−1.3 to 5.4)	0.221	0.541
Sensory onset latency (ms)	−0.17 (−0.35 to 0.02)	0.082	0.290	−0.18 (−0.39 to 0.02)	0.070	0.331
CMAP amplitude, wrist (mV)	−0.14 (−0.95 to 0.66)	0.721	0.914	0.05 (−0.79 to 0.90)	0.898	0.987
CMAP amplitude, below elbow (mV)	0.02 (−0.74 to 0.79)	0.955	0.959	0.19 (−0.63 to 1.02)	0.640	0.869
CMAP amplitude, above elbow (mV)	−0.12 (−0.88 to 0.64)	0.752	0.914	0.10 (−0.69 to 0.90)	0.793	0.928
MCV (m/s)	4.4 (1.0 to 7.9)	0.012	0.104	4.8 (1.1 to 8.5)	0.012	0.165
Motor latency, wrist (ms)	−0.11 (−0.38 to 0.16)	0.406	0.712	−0.05 (−0.34 to 0.24)	0.717	0.891
Motor latency, below elbow (ms)	−0.54 (−0.95 to −0.14)	0.010	0.104	−0.51 (−0.94 to −0.08)	0.022	0.203
Motor latency, above elbow (ms)	−0.66 (−1.11 to −0.21)	0.005	0.074	−0.66 (−1.14 to −0.17)	0.009	0.148
Right radial nerve — Model 3 covariate: forearm circumference						
SNAP amplitude (1st digit) (μV)	−2.68 (−7.21 to 1.85)	0.243	0.520	−3.05 (−7.89 to 1.79)	0.213	0.531
SCV (m/s)	3.4 (−1.0 to 7.9)	0.129	0.405	3.8 (−1.0 to 8.6)	0.115	0.396
Sensory onset latency (ms)	−0.15 (−0.32 to 0.01)	0.071	0.275	−0.16 (−0.34 to 0.02)	0.088	0.372
CMAP amplitude, forearm (mV)	−0.08 (−1.08 to 0.93)	0.881	0.935	−0.20 (−1.26 to 0.86)	0.709	0.891
CMAP amplitude, elbow (mV)	0.49 (−0.21 to 1.19)	0.163	0.418	0.52 (−0.23 to 1.27)	0.169	0.486
MCV (m/s)	1.9 (−2.7 to 6.5)	0.411	0.712	1.8 (−3.1 to 6.7)	0.470	0.795
Motor latency, forearm (ms)	0.03 (−0.27 to 0.33)	0.847	0.923	0.00 (−0.33 to 0.33)	0.997	0.997
Motor latency, elbow (ms)	0.24 (−0.38 to 0.86)	0.441	0.724	0.10 (−0.56 to 0.76)	0.761	0.920
Left median nerve — Model 3 covariate: forearm circumference						
SNAP amplitude (2nd digit) (μV)	−1.49 (−8.42 to 5.45)	0.670	0.896	−2.42 (−10.01 to 5.17)	0.526	0.824
SCV (m/s)	2.9 (−1.1 to 6.9)	0.154	0.414	3.1 (−1.1 to 7.4)	0.147	0.451
Sensory onset latency (ms)	−0.01 (−0.19 to 0.17)	0.892	0.935	−0.05 (−0.24 to 0.14)	0.634	0.869

Parameter	Model 2 β (95% CI)	p	q	Model 3 β (95% CI)	p	q
CMAP amplitude, wrist (mV)	−0.97 (−2.47 to 0.54)	0.204	0.495	−0.83 (−2.48 to 0.81)	0.317	0.645
CMAP amplitude, elbow (mV)	−0.94 (−2.33 to 0.46)	0.185	0.463	−0.83 (−2.36 to 0.69)	0.280	0.601
MCV (m/s)	−0.3 (−2.7 to 2.2)	0.834	0.923	−0.2 (−2.8 to 2.5)	0.909	0.987
Motor latency, wrist (ms)	−0.05 (−0.25 to 0.15)	0.638	0.878	−0.04 (−0.25 to 0.17)	0.718	0.891
Motor latency, elbow (ms)	−0.16 (−0.53 to 0.21)	0.390	0.712	−0.10 (−0.49 to 0.30)	0.620	0.869
Left ulnar nerve — Model 3 covariate: forearm circumference						
SNAP amplitude (5th digit) (μV)	−5.64 (−11.47 to 0.19)	0.058	0.255	−5.28 (−11.71 to 1.15)	0.106	0.396
SCV (m/s)	−0.8 (−3.7 to 2.1)	0.579	0.860	−0.1 (−3.2 to 3.1)	0.966	0.994
Sensory onset latency (ms)	−0.04 (−0.21 to 0.13)	0.626	0.871	−0.10 (−0.28 to 0.08)	0.267	0.592
CMAP amplitude, wrist (mV)	0.07 (−1.00 to 1.13)	0.901	0.935	0.45 (−0.68 to 1.58)	0.429	0.750
CMAP amplitude, below elbow (mV)	−0.21 (−1.22 to 0.81)	0.684	0.896	0.12 (−0.98 to 1.22)	0.826	0.953
CMAP amplitude, above elbow (mV)	−0.24 (−1.23 to 0.74)	0.623	0.871	0.03 (−1.04 to 1.11)	0.949	0.994
MCV (m/s)	3.5 (−0.2 to 7.2)	0.063	0.255	3.4 (−0.6 to 7.4)	0.090	0.372
Motor latency, wrist (ms)	−0.14 (−0.37 to 0.09)	0.235	0.517	−0.11 (−0.36 to 0.13)	0.358	0.678
Motor latency, below elbow (ms)	−0.47 (−0.92 to −0.02)	0.042	0.209	−0.44 (−0.92 to 0.04)	0.072	0.331
Motor latency, above elbow (ms)	−0.53 (−1.00 to −0.07)	0.026	0.177	−0.50 (−1.00 to 0.00)	0.051	0.269
Left radial nerve — Model 3 covariate: forearm circumference						
SNAP amplitude (1st digit) (μV)	−0.90 (−5.28 to 3.48)	0.683	0.896	−0.86 (−5.52 to 3.80)	0.714	0.891
SCV (m/s)	0.5 (−3.2 to 4.2)	0.780	0.919	1.1 (−3.1 to 5.2)	0.609	0.869
Sensory onset latency (ms)	−0.16 (−0.33 to 0.01)	0.061	0.255	−0.15 (−0.32 to 0.03)	0.106	0.396
CMAP amplitude, forearm (mV)	−0.17 (−0.83 to 0.49)	0.605	0.871	−0.04 (−0.74 to 0.66)	0.915	0.987
CMAP amplitude, elbow (mV)	−0.02 (−0.69 to 0.66)	0.959	0.959	0.13 (−0.58 to 0.84)	0.721	0.891
MCV (m/s)	0.3 (−3.9 to 4.5)	0.877	0.935	0.8 (−3.6 to 5.3)	0.710	0.891
Motor latency, forearm (ms)	−0.18 (−0.44 to 0.07)	0.154	0.414	−0.18 (−0.46 to 0.09)	0.194	0.509
Motor latency, elbow (ms)	0.21 (−0.12 to 0.53)	0.215	0.496	0.18 (−0.17 to 0.54)	0.311	0.645

β = adjusted mean difference, football players minus controls, in the units of each parameter. Model 2: age, height and body mass index; Model 3: age and the circumference of the limb segment over which the response was recorded. q, Benjamini–Hochberg FDR across all 110 NCS parameters within each model. Results for Model 1 are given in Supplementary Table S1.

Table S4. Sensitivity of the age-adjusted results (Model 1) to the handling of values that could not be verified against the EMG reports.

NCS parameter	Main analysis β (95% CI)	q	All values as recorded β (95% CI)	q	Stricter exclusion β (95% CI)	q
Right common peroneal nerve: MCV, distal segment (m/s)	3.6 (1.1 to 6.1)	0.087	3.6 (1.1 to 6.1)	0.096	3.6 (1.1 to 6.1)	0.075
Right common peroneal nerve: Motor latency, fibular head (ms)	−0.69 (−1.22 to −0.16)	0.116	−0.69 (−1.22 to −0.16)	0.128	−0.69 (−1.22 to −0.16)	0.106
Right tibial nerve: CMAP amplitude, ankle (mV)	−1.68 (−3.19 to −0.17)	0.182	−1.68 (−3.19 to −0.17)	0.205	−1.68 (−3.19 to −0.17)	0.173
Right tibial nerve: CMAP amplitude, popliteal fossa (mV) †	−1.73 (−3.14 to −0.32)	0.141	−0.64 (−3.79 to 2.52)	0.877	−1.73 (−3.14 to −0.32)	0.131
Right sural nerve: SNAP amplitude (μV)	−3.82 (−7.95 to 0.31)	0.305	−3.82 (−7.95 to 0.31)	0.332	−3.82 (−7.95 to 0.31)	0.294
Left common peroneal nerve: MCV, distal segment (m/s)	2.7 (0.2 to 5.3)	0.194	2.7 (0.2 to 5.3)	0.214	2.7 (0.2 to 5.3)	0.185
Left tibial nerve: CMAP amplitude, ankle (mV)	−1.43 (−3.12 to 0.26)	0.340	−1.43 (−3.12 to 0.26)	0.377	−1.43 (−3.12 to 0.26)	0.330
Left tibial nerve: CMAP amplitude, popliteal fossa (mV)	−1.68 (−3.25 to −0.11)	0.194	−1.68 (−3.25 to −0.11)	0.214	−1.68 (−3.25 to −0.11)	0.185
Left tibial nerve: MCV (m/s)	2.3 (0.1 to 4.6)	0.215	2.3 (0.1 to 4.6)	0.235	2.3 (0.1 to 4.6)	0.206
Left tibial nerve: Motor latency, popliteal fossa (ms)	−0.81 (−1.50 to −0.12)	0.162	−0.81 (−1.50 to −0.12)	0.185	−0.81 (−1.50 to −0.12)	0.144
Left sural nerve: SNAP amplitude (μV)	−4.55 (−8.47 to −0.63)	0.162	−4.55 (−8.47 to −0.63)	0.185	−4.55 (−8.47 to −0.63)	0.144
Left sural nerve: SCV (m/s)	6.9 (3.2 to 10.7)	**0.012**	6.9 (3.2 to 10.7)	**0.016**	6.9 (3.2 to 10.7)	**0.012**
Right accessory nerve: Motor latency (ms) †	1.17 (0.74 to 1.60)	**<0.001**	0.60 (−1.03 to 2.23)	0.761	1.17 (0.74 to 1.60)	**<0.001**
Right axillary nerve: Motor latency (ms)	0.59 (0.16 to 1.02)	0.087	0.59 (0.16 to 1.02)	0.096	0.59 (0.16 to 1.02)	0.079
Right musculocutaneous nerve: Motor latency (ms)	−0.62 (−1.05 to −0.18)	0.087	−0.62 (−1.05 to −0.18)	0.096	−0.62 (−1.05 to −0.18)	0.079
Left accessory nerve: Motor latency (ms)	1.29 (0.89 to 1.69)	**<0.001**	1.29 (0.89 to 1.69)	**<0.001**	1.29 (0.89 to 1.69)	**<0.001**
Left musculocutaneous nerve: Motor latency (ms)	−0.82 (−1.25 to −0.38)	**0.012**	−0.82 (−1.25 to −0.38)	**0.016**	−0.82 (−1.25 to −0.38)	**0.012**
Right median nerve: SNAP amplitude (2nd digit) (μV)	−8.17 (−14.70 to −1.64)	0.137	−8.17 (−14.70 to −1.64)	0.149	−8.17 (−14.70 to −1.64)	0.126
Right ulnar nerve: SNAP amplitude (5th digit) (μV) †	−9.40 (−15.74 to −3.07)	0.087	−9.40 (−15.74 to −3.07)	0.096	−9.94 (−16.16 to −3.71)	**0.040**
Right ulnar nerve: MCV (m/s)	4.0 (0.5 to 7.4)	0.162	4.0 (0.5 to 7.4)	0.185	4.0 (0.5 to 7.4)	0.144
Right ulnar nerve: Motor latency, below elbow (ms)	−0.54 (−0.94 to −0.15)	0.087	−0.54 (−0.94 to −0.15)	0.096	−0.54 (−0.94 to −0.15)	0.079
Right ulnar nerve: Motor latency, above elbow (ms)	−0.62 (−1.07 to −0.18)	0.087	−0.62 (−1.07 to −0.18)	0.096	−0.62 (−1.07 to −0.18)	0.079
Right radial nerve: Motor latency, elbow (ms) †	0.28 (−0.33 to 0.90)	0.668	0.28 (−0.33 to 0.90)	0.694	0.50 (0.21 to 0.78)	**0.020**
Left median nerve: SNAP amplitude (2nd digit) (μV)	−2.43 (−9.42 to 4.56)	0.781	−2.43 (−9.42 to 4.56)	0.793	−2.43 (−9.42 to 4.56)	0.770
Left median nerve: CMAP amplitude, wrist (mV)	−1.21 (−2.75 to 0.32)	0.365	−1.21 (−2.75 to 0.32)	0.398	−1.21 (−2.75 to 0.32)	0.355
Left median nerve: CMAP amplitude, elbow (mV)	−1.12 (−2.53 to 0.30)	0.365	−1.12 (−2.53 to 0.30)	0.398	−1.12 (−2.53 to 0.30)	0.355
Left ulnar nerve: SNAP amplitude (5th digit) (μV) †	−6.73 (−12.71 to −0.75)	0.181	−6.73 (−12.71 to −0.75)	0.205	−7.15 (−13.08 to −1.22)	0.137
Left ulnar nerve: Motor latency, below elbow (ms)	−0.45 (−0.90 to −0.01)	0.215	−0.45 (−0.90 to −0.01)	0.235	−0.45 (−0.90 to −0.01)	0.206
Left ulnar nerve: Motor latency, above elbow (ms)	−0.49 (−0.96 to −0.03)	0.194	−0.49 (−0.96 to −0.03)	0.214	−0.49 (−0.96 to −0.03)	0.185

Model 1 (adjusted for age); β = adjusted mean difference, football players minus controls. q, Benjamini–Hochberg FDR across all 110 NCS parameters within each analysis; $q < 0.05$ in bold. Main analysis: 10 values meeting the exclusion rules set to missing. All values as recorded: no values set to missing. Stricter exclusion: the 12 abnormal but possible unverifiable values were also set to missing (Supplementary Table S2). Rows show all parameters with $p < 0.05$ in the unadjusted or Model 1 analysis in at least one of the three analyses. † Parameter affected by the handling of these values; for all other parameters the β estimates were identical in the three analyses (q-values differ slightly because each FDR adjustment includes all parameters).
